# Comparison of the scavenging capacities of phloroglucinol and 2,4,6-trihydroxypyridine towards HO˙ radical: a computational study†

**DOI:** 10.1039/d0ra08377a

**Published:** 2020-11-27

**Authors:** Žiko Milanović, Jelena Tošović, Svetlana Marković, Zoran Marković

**Affiliations:** Department of Chemistry, Faculty of Science, University of Kragujevac 12 Radoja Domanovića 34000 Kragujevac Serbia jelena.tosovic@pmf.kg.ac.rs; Department of Science, Institute for Information Technologies, University of Kragujevac Jovana Civijića bb 34000 Kragujevac Serbia

## Abstract

In this work the scavenging capacities of biologically active phloroglucinol (1,3,5-trihydroxybenzene, THB–OH) and structurally similar 2,4,6-trihydroxypyridine (THP–OH) towards HO˙ were examined. This task was realized by means of density functional theory, through investigation of all favorable antioxidative pathways in two solvents of different polarity: benzene and water. It was found that in benzene both compounds conform to the hydrogen atom transfer (HAT) and radical adduct formation (RAF) mechanisms. In water, the mechanisms of antioxidative action of the investigated compounds are far more complex, especially those of THB–OH. This compound and HO˙ undergo all four investigated mechanisms: HAT, RAF, sequential proton loss electron transfer (SPLET), and single electron transfer-proton transfer (SET-PT). HAT, RAF and SPLET are operative mechanisms in the case of THP–OH. Independently of solvent polarity, both investigated compounds are more reactive towards HO˙ in comparison to Trolox. Our final remark is as follows: the electron-withdrawing effect of the nitrogen is stronger than the electron-donating effect of the OH groups in the molecule of THP–OH. As a consequence, THB–OH is more powerful antioxidant than THP–OH, thus implying that the presence of nitrogen decreases the scavenging capacity of the respective compound.

## Abstract 2

The scavenging capacities of biologically active phloroglucinol (1,3,5-trihydroxybenzene, THB–OH) and structurally similar 2,4,6-trihydroxypyridine (THP–OH) towards HO˙ were elucidated.

## Graphical abstract

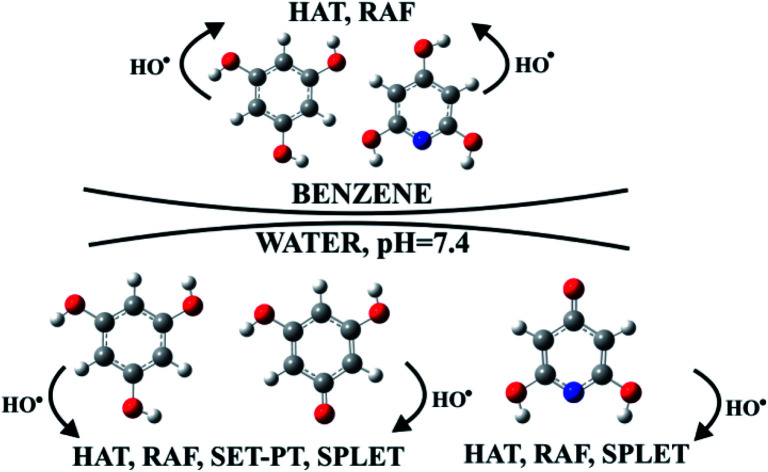

## 1. Introduction

During normal metabolic functions, highly reactive oxygen and nitrogen species are generated in our organism.^1^ However, if the production of these reactive species is increased, the pro-oxidant–antioxidant balance in the cells is shifted towards the pro-oxidants.^2^ This state is known as oxidative stress. Oxidative stress causes many pathogenic processes including cancer, ageing, rheumatoid arthritis, and inflammation amongst the others.^3–5^ Among all oxygen-centred radicals which can cause oxidative stress, HO˙ is the most reactive.^6^ Due to its high reactivity, the selectivity of the HO˙ radical is very low. It can react with a vast number of chemical compounds through a wide variety of mechanisms. It has been reported that HO˙ is responsible for 60–70% of the tissue damage affected by ionizing radiations.^7^ Furthermore, it has been estimated that it is responsible for the crucial oxidative damage to DNA.^8^

Edible seaweeds serve as a rich source of structurally diverse compounds which can protect cells from oxidative stress by eliminating free radicals.^9,10^ Among them, phloroglucinol (1,3,5-trihydroxybenzene, THB–OH), a component of phlorotannins, can be most abundantly found in edible brown alga *Ecklonia cava*.^11,12^ It is also present at high levels in other brown algae *Ishige okamurae*, *Fucaceae*, as well as in *Himanthalia elongate*, consumed as “Sea spaghetti” in France and Ireland.^13–15^ Along with its derivatives, THB–OH belongs to a class of phenolic compounds which are considered as semi-essential nutrients for human diet.^16^ It has been shown that THB–OH induces beneficial health effects by scavenging oxygen-centred radicals, inhibiting apoptosis, and protecting cells against oxidative stress.^12,17–23^ Due to strong antioxidative activity, it has a variety of uses in pharmacology.^24^ For example, THB–OH is known to block apoptosis of lung fibroblasts. Apoptosis of lung fibroblasts and keratinocytes, along with damage of mouse skin that is induced by UV radiation, is counteracted by restoring the activity of antioxidant enzymes associated with the elimination of reactive oxygen species (ROS) production when THB–OH is introduced.^25,26^ In addition, apoptosis of lung fibroblasts, splenocytes and blood lymphocytes, induced by ionizing radiation is blocked by inhibition of the production of intracellular ROS by this compound.^27,28^ Furthermore, apoptosis and DNA damage that are induced by oxidative stress can be counteracted by the protection of HaCaT human keratinocytes by THB–OH.^29^ Moreover, THB–OH has broad therapeutic effects, including antimicrobial, antiallergy, antispasmodic, anti-inflammatory, antidiabetic, and anticarcinogenic^12,13,30–33^ influences. In addition, it is extensively utilized for the treatment of gastrointestinal disorders such as spasmodic pain or gallstones.^34^ Finally, THB–OH and its derivatives have been successfully applied in urology for the treatment of nephritic colic and as an anti-spasmodic agents, as a dermatological products, and for muscle relaxation.^24,35–37^

2,4,6-Trihydroxypyridine (THP–OH) is structurally similar to THB–OH, where THP–OH contains a nitrogen atom in the aromatic ring. It has been reported in the literature that THP–OH may possess antineurotic properties.^38^

To the best of our knowledge, theoretical studies of the mechanisms of the antioxidative action of these two structurally similar compounds have not been carried out so far. Considering that a molecule of THP–OH contains an electron-withdrawing heteroatom and electron-donating phenolic groups, it is interesting to explore the influence of these two opposing effects on scavenging capacity towards hydroxy radical (HO˙). An indicative way to accomplish this aim is to examine and compare antioxidative activities and underlying mechanisms of THB–OH and THP–OH. For this purpose, the reactions between the two selected phenols with HO˙ in non-polar and polar solvents (benzene and water at physiological pH) were investigated using the quantum mechanics-based test for overall free-radical scavenging activity protocol. An additional goal was to investigate the antioxidative activity of THB–OH and THP–OH relative to a reference compound, Trolox (6-hydroxy-2,5,7,8-tetramethylchroman-2-carboxylic acid).

## 2. Materials and methods

### 2.1. General details

Within this work, all calculations were performed by the Gaussian 09 program package.^39^ The hybrid meta functional M06-2X in conjunction with the 6-311++G(d,p) basis set^40^ and CPCM polarizable continuum solvation model were used for the optimisation of all structures without any geometrical constraints.^41^ Benzene (dielectric constant, *ε* = 2.2706) and water solutions (*ε* = 78.3553) were employed to mimic non-polar and polar environments. Previous studies have shown that the applied theoretical model is suitable for thermochemical and kinetic research of similar issues.^42–45^ By analysing the results of the frequency calculations, the nature of the stationary points was revealed: one imaginary frequency for transition states and no imaginary frequencies for equilibrium geometries. The intrinsic reaction coordinate (IRC) calculations were additionally performed to confirm the validity of each transition state. The Gibbs free energies of the examined reactions (Δ_r_*G*) were determined at standard conditions (*T* = 298.15 K and *P* = 101 325 Pa).

### 2.2. QM-ORSA protocol

Likewise in experimental research, it is possible to determine relative antioxidative capacity by theoretical means, using the protocol called Quantum Mechanics-based test for Overall free-Radical Scavenging Activity (QM-ORSA).^46^ This protocol involves the evaluation of thermodynamic and kinetic parameters for all favourable reaction pathways of both antioxidant and reference compounds (usually Trolox, Tx). It also takes into account different reaction conditions, such as solvent polarity and pH. Another advantage of the QM-ORSA protocol is that it provides a relative amount of products and predicts dominant mechanistic pathways.^46^ It is very challenging to obtain these data through experiments. It should be pointed out that this methodology has been successfully applied in the scientific literature for the investigation of primary and relative antioxidative activities.^44,45,47–54^

### 2.3. Calculation of the rate constants

Hydrogen atom transfer (HAT), radical adduct formation (RAF), sequential proton loss electron transfer (SPLET), and single electron transfer-proton transfer (SET-PT) mechanisms were investigated for the reactions of THB–OH and THP–OH with HO˙ radical. All examined reactions are bimolecular.^55^ In the case where a transition state (TS) exists between the reactants and products (HAT and RAF), the rate constants were calculated for 1 M standard state using the conventional transition state theory (TST),^56^ and Eckart method (ZCT-0).^57^ For this purpose, the TheRate program was utilized.^58^ The energy values and partition functions were taken from the quantum mechanical calculations. It was found that several reaction pathways occur without passing through transition states, *i.e.* spontaneously. In such cases dependence of total energy on corresponding scan coordinate (*e.g.* HO˙–X distance) was investigated. For this purpose, HO˙ was located in the vicinity of the X atom and then moved forward to the active centre up to the formation of products. Based on the continuous decrease of total energy with decreasing HO˙–X distance, it was concluded that these reactions are diffusion-controlled, and they were assigned the rate constant of diffusion (1.91 × 10^9^ M^−1^ s^−1^).

In the case of the SET-PT and SPLET mechanisms, which involve a transfer of an electron from a chemical species (*i.e.* atom, molecule, free radical, ion, *etc.*) to another one, transition states do not exist. In these cases, the Marcus theory was used to estimate the activation energies and rate constants.^43,59^

Antioxidative capacity *r*^T^ of the examined antioxidant relative to Tx was calculated using the formula:1
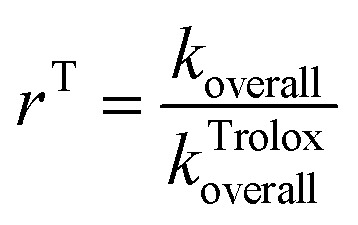


Estimation of the *k*_overall_ and *k*^Trolox^_overall_ values have been comprehensively explained in the literature.^47–49^

The relative amounts of products (%), *i.e.* branching ratios (*Γ*_i_) were determined:2
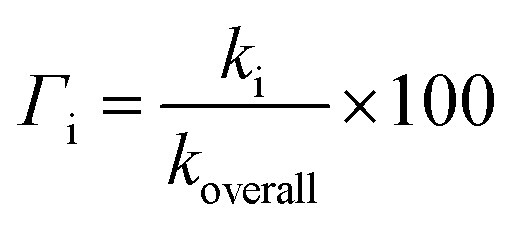
to evaluate which of the mechanistic pathways, i, is dominant.

## 3. Results and discussion

### 3.1. Thermodynamic approach

**Fig. 1:**
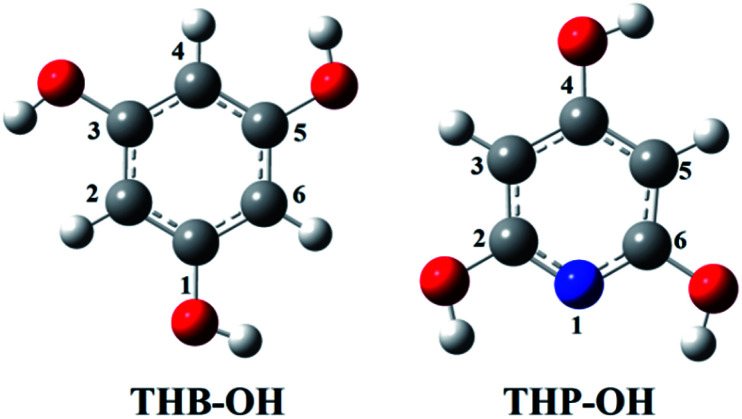
Optimized geometries of the most stable rotamers of the investigated compounds in benzene. An atom labelling scheme is provided (grey – carbon atoms, white – hydrogen atoms, red – oxygen atoms, blue – nitrogen atoms).

Only the neutral forms of the investigated compounds are present in non-polar solvents. The optimized structures of THB–OH and THP–OH in benzene are shown in Fig. 1. These structures were further used for investigation of the antioxidative mechanisms. A tautomer of THP–OH, characterized with a single N1–H2 (N1–H6) bond and double C2=O2 (C6=O6) bond, as well as that characterized with a single N1–H4 bond and double C4=O4 bond, are by around 13 and 45 kJ mol^−1^ less stable than the structure depicted in Fig. 1. For this reason, these tautomers of THP–OH were not further considered.

To investigate the scavenging capacities of the selected compounds towards HO˙ radical in benzene the following reactions were examined:

3 HAT: Ar–OH + HO˙ → Ar–O˙ + H_2_O

4 RAF: Ar–OH + HO˙ → [HO–Ar–OH]˙

5 SET: Ar–OH + HO˙ → Ar–OH˙^+^ + HO^−^

6 PT: Ar–OH˙^+^ + HO^−^ → Ar–O˙ + H_2_O

7b SPL: Ar–OH → Ar–O^−^ + H^+^

8 ET: Ar–O^−^ + HO˙ → Ar–O˙ + HO^−^

where Ar–OH, Ar–O˙, (HO–Ar–OH)˙, Ar–OH˙^+^, and Ar–O^−^ denote antioxidant, its radical, radical adduct, radical cation, and anion, respectively. In the SPL reaction b refers to benzene solution.

**Fig. 2:**
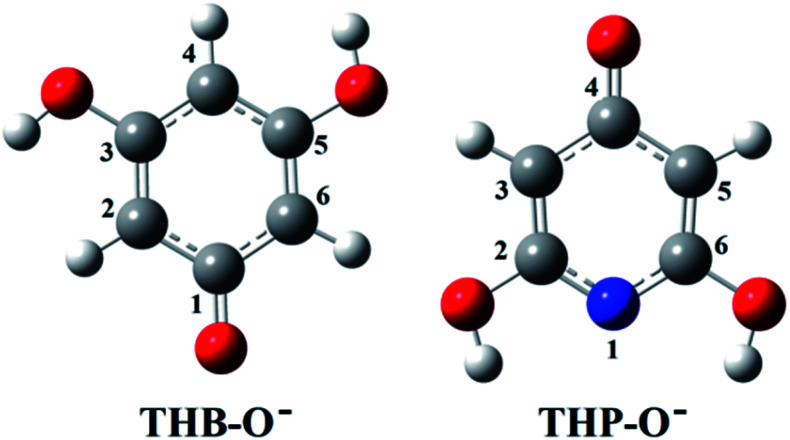
Optimized geometries of the most stable rotamers of the monoanions of the investigated compounds in water. An atom labelling scheme is provided.

Investigation of the antioxidative mechanisms in aqueous solution is far more complicated. First, it is necessary to evaluate which acid–base forms of the two compounds are dominant in the aqueous solution at the physiological pH = 7.4. Based on the p*K*_a_ values of THB–OH and THP–OH the molar fractions of the acid–base forms (neutral molecules, monoanions, dianions, and fully deprotonated molecules) were estimated. The experimental p*K*_a_ values^60,61^ and deprotonation routes of the investigated compounds are shown in Fig. S1 (ESI).† The dominant acid–base forms of THB–OH are neutral and monoanionic (79.7% and 20.0%). The dominant species of THP–OH is monoanionic with a population of 97.4%, whereas the population of the dianion is negligible (2.5%). The scavenging capacity was investigated for all dominant species. The optimized structures of the two monoanions in water solution are presented in Fig. 2.

The reaction pathways for the neutral form of THB–OH in water are similar to those in a non-polar solvent (reactions (3)–(8)). The only difference is in the first step of the SPLET mechanism (reaction (7)). Namely, it is reasonable to assume that a certain amount of HO^−^ is present in basic aqueous solution, and therefore this reaction is examined as:

7w ArOH + HO^−^ → ArO^−^ + H_2_O

where w refers to water solution.

As for the monoanions, reaction pathways for THB–O^−^ are very complex. Note that THB–O^−^ is formed in the second step of the SPLET mechanism of the parent molecule (reaction (8) in aqueous solution). In addition, there is no clear boundary between the SPLET and SET-PT mechanisms of the monoanion; they intertwine. These facts are of particular importance for understanding the behaviour of THB–OH and its monoanion under physiological conditions. Therefore, the reactions associated with antioxidative activity of THB–O^−^ in water are as follows:

9 SET: THB–O^−^ + HO˙ → THB–O˙ + HO^−^

10 PT: THB–O˙ + HO^−^ → THB–O˙^−^ + H_2_O

11 ET: THB–O˙^−^ + HO˙ → THB–O˙˙ + HO^−^

12 HAT: THB–O˙ + HO˙ → THB–O˙˙ + H_2_O

13 RAF: THB–O˙ + HO˙ → HO–THBO

On the other hand, behaviour of THP–O^−^ can be simply described with the following reactions:

14 HAT: THP–O^−^ + HO˙ → THP–O˙^−^ + H_2_O

15 RAF: THP–O^−^ + HO˙ → [HO–THP–O]˙^−^

16 SET: THP–O^−^ + HO˙ → THP–O˙ + HO^−^

17 PT: THP–O˙ + HO^−^ → THP–O˙^−^ + H_2_O

18 SPL: THP–O^−^ + HO^−^ → THP–O^2−^ + H_2_O

19 ET: THP–O^2−^ + HO˙ → THP–O˙^−^ + HO^−^

To select exergonic reaction pathways for further kinetic investigations the Gibbs free energies for all antioxidative reaction pathways in benzene and water (reactions (3)–(19)) were calculated and summarized in Table 1. According to the QM-ORSA protocol, both reaction steps in two-step antioxidative mechanisms (SET-PT and SPLET) need to be exergonic to be considered for kinetic investigations.^46^

**Table 1:** Reaction free energies, Δ_r_*G* (kJ mol^−1^)

Position	RAF	Position	HAT	SET-PT		SPLET	
	Δ_r_*G*		Δ_r_*G*	Δ_r_*G*_1_	Δ_r_*G*_2_	Δ_r_*G*_1_	Δ_r_*G*_2_
**Benzene (*ε* = 2.2706)**							
*THB–OH*							
1-C, 3-C, 5-C	−28.7	1-OH, 3-OH, 5-OH	−118.7	357.3	−476.1	432.4	21.7
2-C, 4-C, 6-C	−39.0						
*THP–OH*							
2-C	−32.2	2-OH	−97.7	375.5	−473.6	419.6	55.5
3-C	−27.4	4-OH	−35.9		−392.4	402.4	153.9
4-C	2.6						
5-C	−32.0	6-OH	−99.0		−475.0	415.7	58.1
6-C	−32.3						

**Water (*ε* = 78.3553)**							
*THB–OH*							
1-C, 3-C, 5-C	−26.7	1-OH, 3-OH, 5-OH	−124.5	130.5	−255.0	−94.3	−30.3
2-C, 4-C, 6-C	−34.5						
*THB–O^−^*							
2-C	−246.7	3-OH, 5-OH	−131.8	−30.3	−125.7	−125.7	−6.2
4-C	−240.7						
6-C	−242.4						
*THP–O^−^*							
2-C, 6-C	−32.4	2-OH, 6-OH	−134.6	87.8	−222.4	−63.3	−71.3
3-C, 5-C	−33.8						
4-C	51.6						

**Fig. 3:**
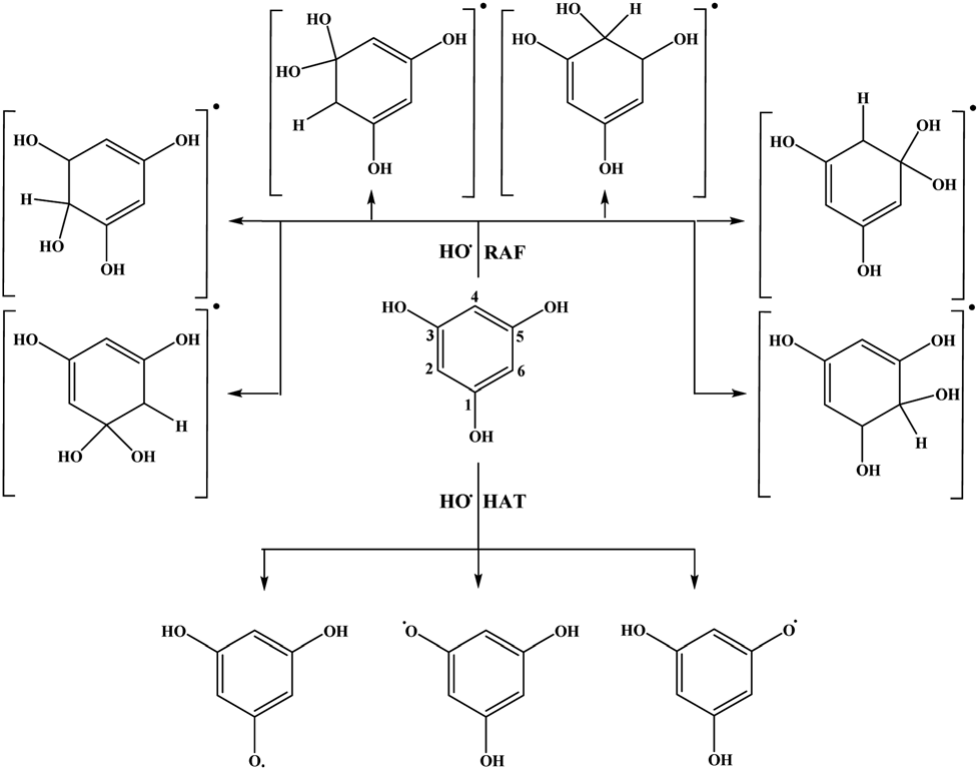
Favourable antioxidative reaction pathways of THB–OH in benzene.

**Fig. 4:**
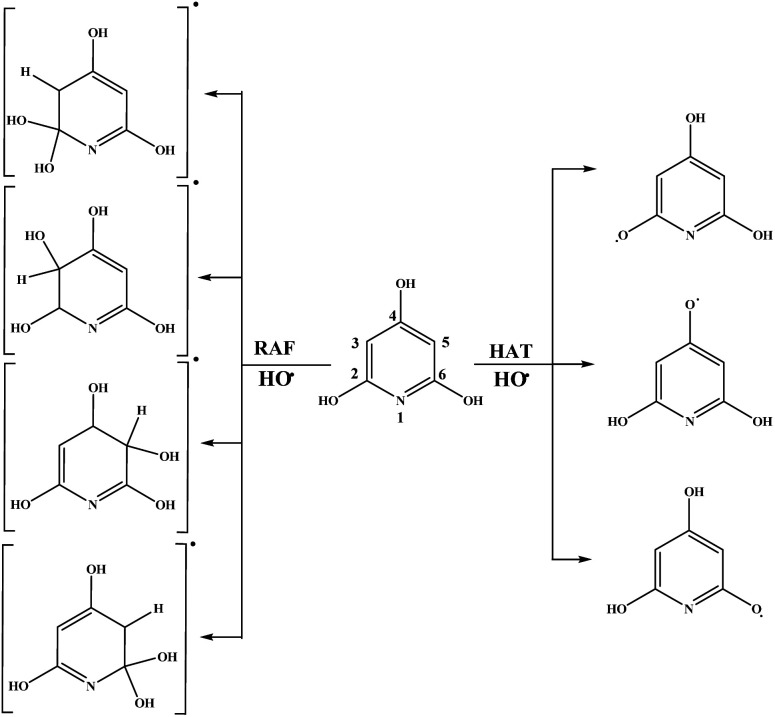
Favourable antioxidative reaction pathways of THP–OH in benzene.

The results related to the Δ_r_*G* values in the benzene solution will be discussed first. Schematic representation of all favourable reaction pathways is presented in Fig. 3 and 4. Representative graphical models related to these reaction paths are depicted in Fig. S2 and S3.† As expected, highly endergonic first steps of the SET-PT and SPLET mechanisms indicate that these reaction pathways are not plausible for both compounds (Table 1).

On the other hand, HAT and RAF are in general thermodynamically favourable reaction paths because more stable radicals are obtained as the products of these reactions. The reactions for THP–OH are mainly less exergonic than those for THB–OH; an RAF pathway for THP–OH is even slightly endergonic (the 4-C position). These results lead to the following conclusion: the electron-withdrawing effect of the nitrogen is stronger than the electron-donating effect of the OH groups, which leads to decreased electron density in the aromatic ring of THP–OH in comparison to THB–OH. Consequently, the reactivity of THP–OH towards the HO˙ radical in electrophilic addition (RAF) and hydrogen atom abstraction (HAT) is weaker than that of THB–OH.

All positions in THB–OH are susceptible to the addition of the electrophilic HO˙ radical, whereas the behaviour of the *ortho*, *meta*, and *para* positions of THP–OH is unusual. Namely, the *ortho* and *meta* carbon atoms are favourable for an electrophilic attack, whereas the *para* carbon atom and nitrogen are not. These findings can be explained with the shapes of the HOMOs of the two molecules (Fig. S2 and S3†). THB–OH has two degenerate HOMOs that cover the whole aromatic ring, while the greatest contribution to the HOMO of THP–OH comes exactly from the *ortho* and *meta* carbons. The HAT pathways in the *ortho* positions of THP–OH are more exergonic than that in the *para* positions. A reason for this occurrence can be better delocalization of the unpaired electron where the nitrogen atom is also included (Fig. S3†).

**Fig. 5:**
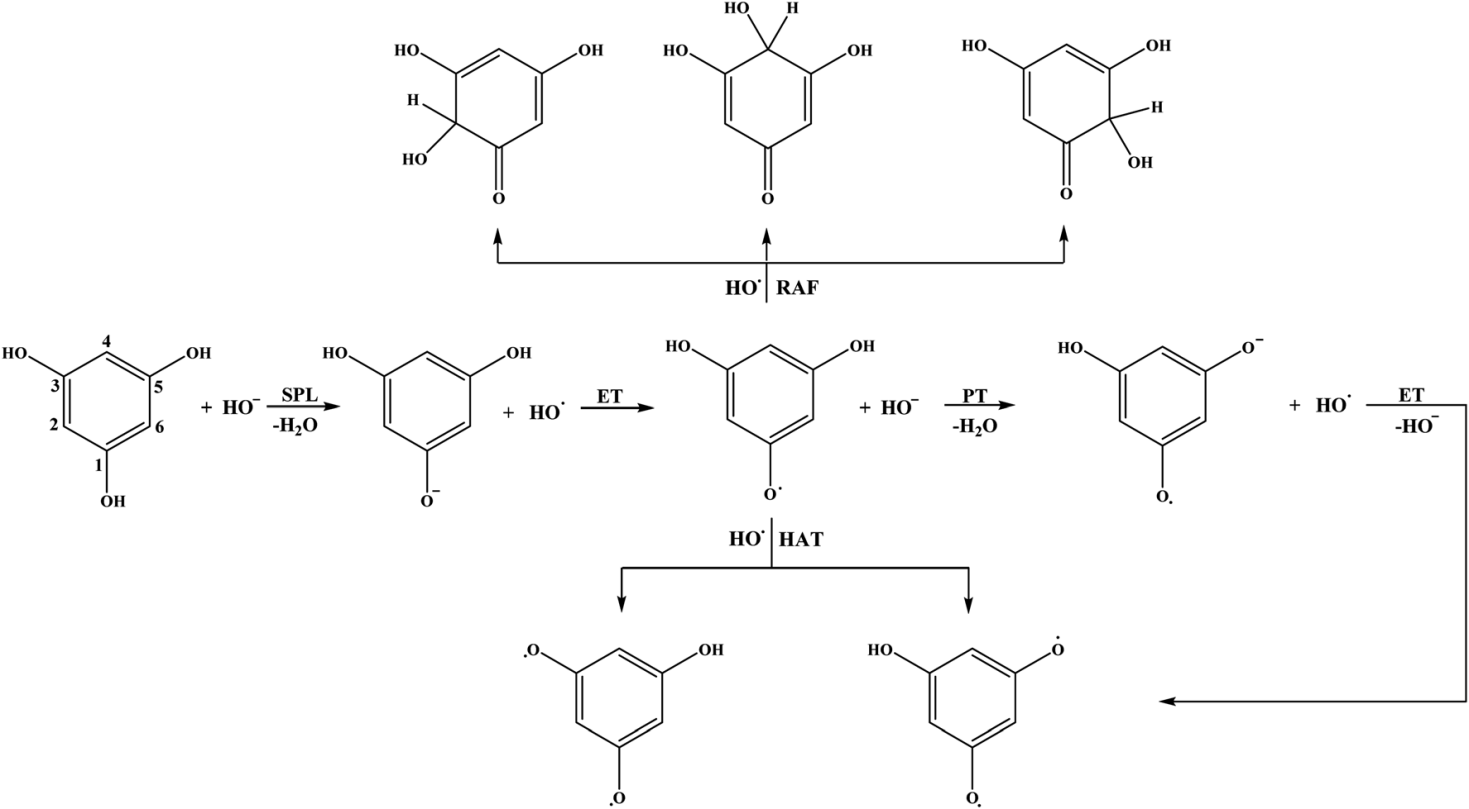
Favourable antioxidative reaction pathways of THB–OH in water.

**Fig. 6:**
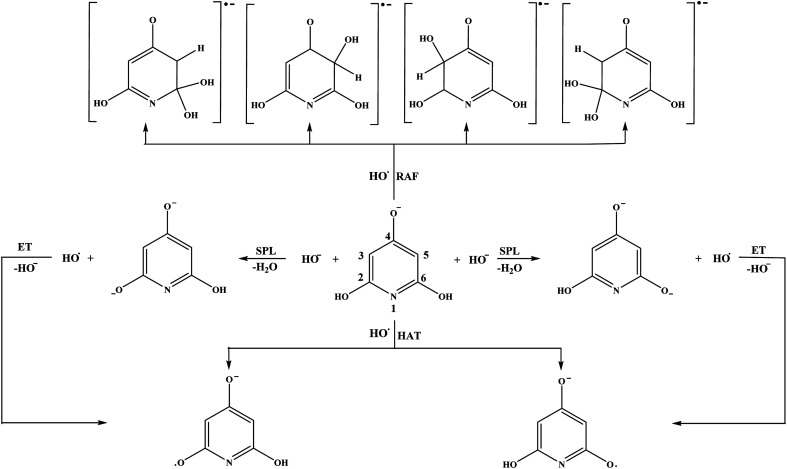
Favourable antioxidative reaction pathways of THP–O^−^ in water.

Schematic representations of all favourable reaction pathways in water for neutral and monoanionic species of the investigated compounds are depicted in Fig. 5 and 6.

As for the behaviour of THB–OH in a slightly basic aqueous solution, only SET-PT is not a plausible reaction path. As expected, the Δ_r_*G* values for the RAF and HAT pathways in water are comparative to those in benzene, and the presence of HO^−^ enables deprotonation of THB–OH (reaction (7w)), where the THB–O^−^ anion is formed.

Very complex behaviour of THB–O^−^ in water solution (reactions (9)–(13)) is worth discussion. In the presence of HO˙, THB–O^−^ spontaneously donates an electron to HO˙, thus yielding THB–O˙ and HO^−^. The higher HOMO energy of THB–O^−^ (−0.2240 au) in comparison to the SOMO energy of HO˙ (−0.4423 au) enables this exergonic electron transfer reaction (Fig. S4†). Further steps: the transformation of THB–O˙ and HO^−^ into THB–O˙^−^ and H_2_O, and transformation of THB–O˙^−^ and HO˙ into THB–O˙˙ and HO^−^, are also exergonic. One can conclude that, in a basic water environment, THB–O^−^ and HO˙ undergo both SPLET and SET-PT mechanisms. It should be pointed out that this is one of the rare cases where the SET-PT mechanism is operative. The finding that THB–O^−^ does not exist in the presence of HO˙, as it spontaneously converts to THB–O˙, suggests that HAT and RAF should be examined as the reactions of THB–O˙ with HO˙. It is noticeable that the RAF pathways of THB–O˙ in the 2-C, 4-C, and 6-C positions are the most exergonic reactions in Table 1. As the spin density distribution of THB–O˙ in water is practically identical to that in benzene (Fig. S2†), it is apparent that the unpaired electron is delocalized exactly over the 2-C, 4-C, and 6-C atoms, implying that these particular atoms are susceptible to addition of free radicals. Some intermediates and products of the reactions of THB–O^−^ with HO˙ in aqueous solution are presented in Fig. S5.†

Based on the Δ_r_*G* values it is clear that THP–O^−^ does not undergo the SET-PT mechanism (Fig. S6†), whereas HAT and SPLET are plausible mechanistic pathways. The behaviour of THP–O^−^ in the RAF pathways is similar to that of the parent molecule in benzene (Table 1): it reacts with HO˙ in the *ortho* and *meta* positions to yield radical anions of almost identical stability, whereas the *para* carbon and nitrogen are not susceptible for the electrophilic attack of HO˙. Some intermediates and products of the reactions of THP–O^−^ with HO˙ in aqueous solution are presented in Fig. S7.†

If we compare the reactivity of the two anions towards HO˙, we will come to the same conclusion: THB–O^−^ is more reactive than THP–O^−^, as it undergoes all antioxidative pathways. Certainly, we need to keep in mind that THB–O^−^ spontaneously transforms into THB–O˙, which is also a manifestation of its reactivity.

### 3.2. Kinetic approach

**Fig. 7:**
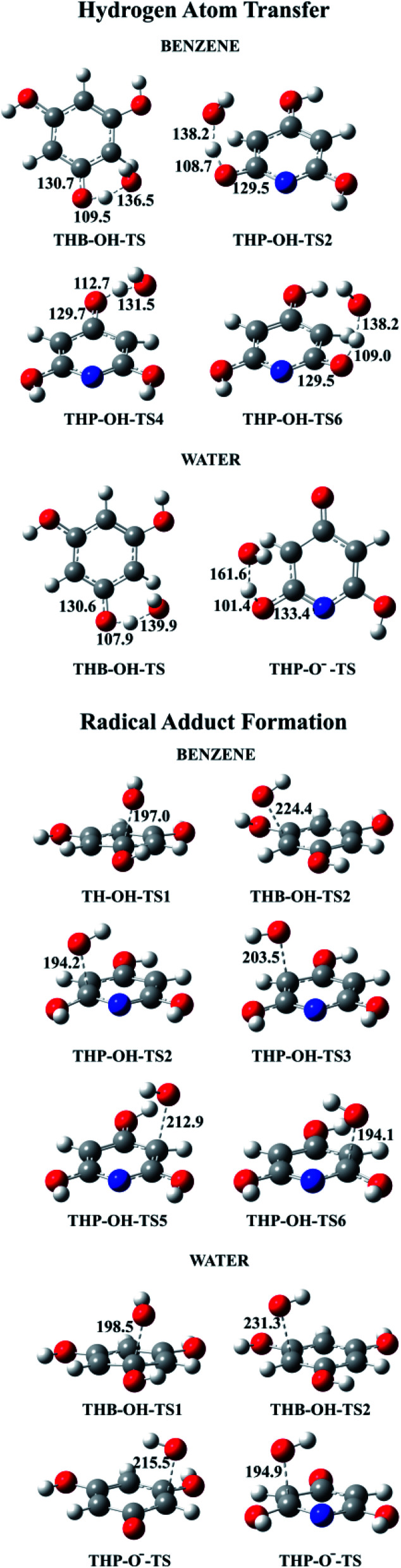
Optimized geometries of all revealed transition states for the HAT and RAF reaction pathways in benzene and water solutions.

All TSs in benzene and majority of TSs in water were identified for thermodynamically favourable HAT and RAF reaction pathways (Fig. 7). Because of symmetry, one TS for three equivalent HAT reactions and two TSs for four RAF reactions of THB–OH in both solutions were determined. In addition, one transition state was obtained for both: two equivalent HAT pathways and two RAF pathways of THP–O^−^. The Gibbs activation energies, as well as rate constants, were calculated. The obtained results are presented in Table 2. Dependence of the rate constants on temperature for the representative HAT and RAF reaction pathways is illustrated with Fig. S8 and S9,† accompanied by a brief discussion.

**Table 2:** Activation energies Δ*G*^‡^_a_ (kJ mol^−1^) and rate constants *k* (M^−1^ s^−1^) for the reactions of THB–OH and THP–OH, as well as their monoanions, with HO˙

Benzene			Water		
Position	Δ*G*^≠^_a_	*k*	Position	Δ*G*^≠^_a_	*k*
**HAT**					
THB–OH			THB–OH		
1-OH, 3-OH, 5-OH	34.0	3.65 × 10^7^	1-OH, 3-OH, 5-OH	35.5	3.65 × 10^7^
			THB–O^−^		
			3-OH, 5-OH	∼0.0	1.91 × 10^9^
THP–OH			THP–O^−^		
2-OH	48.5	9.24 × 10^5^	2-OH, 6-OH	7.8	3.80 × 10^7^
4-OH	42.3	1.45 × 10^7^			
6-OH	45.9	2.56 × 10^6^			

**RAF**					
THB–OH			THB–OH		
1-C, 3-C, 5-C	51.7	1.91 × 10^5^	1-C, 3-C, 5-C	54.5	1.91 × 10^5^
			2-C, 4-C, 6-C	20.5	7.18 × 10^7^
2-C, 4-C, 6-C	21.5	7.18 × 10^7^	THB–O^−^		
			2-C, 4-C, 6-C	∼0.0	1.91 × 10^9^
THP–OH			THP–O^−^		
2-C	58.2	1.43 × 10^4^	2-C, 6-C	33.4	2.94 × 10^7^
3-C	31.5	9.95 × 10^7^			
5-C	25.7	1.12 × 10^8^	3-C, 5-C	∼0.0	1.91 × 10^9^
6-C	58.9	1.06 × 10^4^			

**Electron transfer (ET)**					
—	—	—	THB–O^−^	9.2	7.63 × 10^9^
—	—	—	THB–O˙^−^	0.6	7.94 × 10^9^
—	—	—	THP–O^−^	31.3	2.06 × 10^7^

A common feature of TSs occurring in the HAT reactions is a significant deviation of the phenolic hydrogen atoms, the targets of the HO˙ attack, from planarity (Fig. 7). Similarly, in TSs revealed for the RAF reaction pathways, deviation from planarity of the phenolic –OH groups attached to the C atom exposed to the HO˙ attack was observed (Fig. 7).

Our numerous attempts to locate TSs for the HAT and RAF reactions of THB–O˙ (reactions (12) and (13), and Fig. 5), as well as the RAF reaction pathway in the 3-C (5-C) position of THP–O^−^ (reaction (15) and Fig. 6) were unsuccessful. Considering a pronounced exergonicity of the mentioned reactions (Table 1), it is reasonable to suppose that these processes are spontaneous. To prove this assumption every single reaction was further carefully investigated.

The reactants in the HAT reaction pathway between THB–O˙ and HO˙ are in the triplet state, whereas the products can exist in either singlet or triplet state, depending on the nature of the formed product (THB–O˙˙ or THB=O). For this reason, the stability of the product was investigated in the singlet and triplet ground state. Surprisingly, it was found that the THB–O˙˙ diradical is by 93.2 kJ mol^−1^ more stable than the singlet molecule. A probable reason for this occurrence is a pronounced deviation of the bond lengths and angles in THB=O from those that are common for sp^2^ hybridized atoms (Fig. S10 and Table S1†). It should be pointed out that this is one of the rare cases where the triplet ground state diradical is more stable in comparison to the singlet molecule. Therefore, starting from the triplet reactants the triplet products are formed. Considering this fact, the reaction is further examined in a manner explained in Materials and methods. The HO–H3(H5) distance was selected as a scan coordinate. The continuous decrease of energy going from reactants to products proved the assumption about the spontaneity of this reaction (Fig. S11†).

**Fig. 8:**
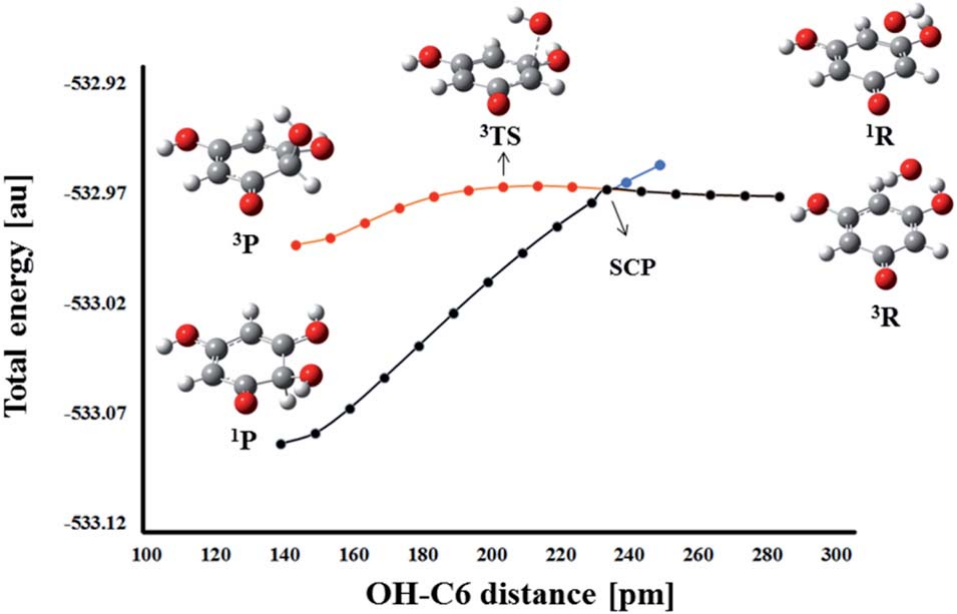
Energy profiles for the RAF pathways of THB–O˙ with HO˙ in the singlet (blue line) and triplet (red line) states. The black line indicates the suggested reaction pathway. The spin crossing point (SCP) appears at the HO–C6 distance of 236.2 pm.

On the other hand, a change in the spin multiplicity along the reaction coordinate occurs in the RAF reaction pathway. Namely, as in the HAT reaction, the reactants THB–O˙ and HO˙ form a triplet, but the respective product HO–THBO is in the singlet state. It is obvious that a simple procedure suitable for the majority of HAT reactions is not applicable to this process. For this particular reaction pathway, that takes place on two potential energy surfaces, the phenomenon called two-state reactivity (TSR),^62,63^ was investigated. This phenomenon has been observed in numerous organic, inorganic, and organometallic reactions,^64,65^ and for the first time has been recently employed in the examination of antioxidative mechanisms.^44,66^ The phenomenon involves the participation of spin-crossing effects for the elucidation of rate constants, branching ratios and reaction mechanisms. Radical adduct formation in the position 6-C of THB–O˙ was selected as representative of the RAF pathways, and the HO–C6 distance was chosen as the scan coordinate. Dependence of total energy on the corresponding scan coordinate was investigated in the following way. The approaching of HO˙ towards the active centre was investigated in the singlet and triplet states (Fig. 8).

In the singlet state, continuous energy decrease going from the reactants to the products was observed. On the other hand, in the triplet spin state, the reaction occurs over a transition state. The reactants are more stable in the triplet state, but the singlet products are far more stable than the triplet products. Therefore, when THB–O˙ and HO˙ are far enough apart, they exist as doublets. As they approach each other, the energy increases slightly to the spin crossing point (SCP). Here, singlet and triplet structures of very similar geometry are almost degenerate (Fig. S12†), which allows spin inversion to take place. Thus, instead of going through the energy-demanding triplet TS, the reaction participants are spontaneously converted into much more stable singlet products. Taking these facts into account the RAF reactions of THB–O˙ with HO˙ need to be considered as diffusion controlled.

In the RAF reaction pathway between THP–O^−^ and HO˙ the spin multiplicity remains the same along the reaction coordinate. The HO–C3(C5) distance was selected as a scan coordinate and based on the continuous decrease of total energy with decreasing of chosen distance, it was concluded that this RAF reaction is diffusion-controlled (Fig. S13†).

As for the proton transfer from THB–OH to HO^−^ (reaction (7w)), and from THB–O˙ to HO^−^ (reaction (10)), the HO–H1(3,5) and HO–H3(5) distances were selected as scan coordinates. Fig. S14† illustrates the barrierless formation of the corresponding products: THB–O^−^ + H_2_O, and THB–O˙^−^ + H_2_O, respectively. The rate constant values of 7.63 × 10^9^ and 7.94 × 10^9^ M^−1^ s^−1^ for the electron transfer reactions from THB–O^−^ to HO˙ (reaction (8) in water solution) and THB–O˙^−^ to HO˙ (reaction (11)) indicate that these reactions are also diffusion controlled.

Based on the continuous decrease of total energy with decreasing of HO–H2(H6) distance in the first step of the SPLET mechanism between THP–O^−^ and HO^−^ (reaction (18) and Fig. S15†), it was concluded that this reaction is also spontaneous, with the rate constant of 1.91 × 10^9^ M^−1^ s^−1^. The second step of the SPLET mechanism, *i.e.* the electron transfer (reaction (19)), requires careful investigation. Namely, for moderately exergonic reactions (such as electron transfer reactions operative in the case of THB–OH and its monoanion) *k*_ET_ increases with the decreasing energy of activation. However, when the reaction becomes highly exergonic, Δ*G*^‡^_a_ surprisingly starts to increase, and consequently, the *k*_ET_ value decreases. This phenomenon, known as an inverted region, was for the first time predicted by Marcus,^59^ and later experimentally confirmed by Miller *et al.*^67^ The electron transfer reaction from THP–O^2−^ to HO˙ is far more exergonic in comparison to the other two electron transfer reactions (Table 1), and the resulting rate constant value is by two orders of magnitude smaller (2.06 × 10^7^ M^−1^ s^−1^). To our best knowledge, this reaction is the first case of inverted-region electron transfer involved in an antioxidative mechanism.

### 3.3. Relative antioxidative activity

Based on the rate constant values calculated for all favourable antioxidative pathways *k*_overall_ values for all investigated compounds, as well as *r*^T^ values were obtained, and the results are presented in Table 3. The *k*^Trolox^_overall_ values of 1.31 × 10^8^ and 1.94 × 10^9^ for benzene and water solutions, respectively, necessary for the obtaining of the relative antioxidative activity, were taken from the literature.^44,45^

**Table 3:** The *k*_overall_ (M^−1^ s^−1^) and *r*^T^ values for the reactions of THB–OH and THP–OH

Compound	Benzene		Water	
	*k*_overall_	*r* ^T^	*k*_overall_	*r* ^T^
THB–OH	3.26 × 10^8^	2.60	7.76 × 10^9^	4.00
THP–OH	2.29 × 10^8^	1.80	5.92 × 10^9^	3.05

According to the *r*^T^ values it is obvious that both THB–OH and THP–OH show higher antioxidative activity in comparison to Tx. THB–OH is a better scavenger of HO˙ than THP–OH, and the efficacy increases with the solvent polarity. A kinetic investigation is in agreement with thermodynamic finding and confirms that the presence of an electron-withdrawing atom in a compound decreases its scavenging capacity.

To evaluate which of the mechanistic pathways is dominant for both investigated compound the branching ratios for benzene and water solutions were calculated (Table S2†). In the case of benzene solution, RAF in the positions 2-C, 4-C, and 6-C (22.0% each), and HAT in the position 1-OH, 3-OH and 5-OH (11.2% each) are the mechanistic pathways involved in the HO˙ scavenging activity of THB–OH. On the other hand, the scavenging activity of THP–OH takes place almost exclusively *via* the RAF mechanism, with the branching ratios of 43.4 and 48.8% for the radical adduct formation reactions in the positions 3-C and 5-C, respectively.

The mechanistic pathways through which THB–OH exerts scavenging activity in water solution completely diverge from those in non-polar benzene. Namely, the main mechanisms involved are SPLET of THB–OH, as well as SET-PT and SPLET mechanisms of its monoanion. As for THP–OH, the RAF and SPLET mechanisms are competitive mechanistic pathways for scavenging the HO˙ radical.

To our best knowledge, there are no experimental literature data related to the rate constants for the reactions of HO˙ with either THB–OH or THP–OH in basic environment. However, there is a valuable experimental work of Wang *et al.* where the reactions of THB–OH with the HO˙, ˙N_3_, and Br_2_˙^−^ radicals were examined in acidic and basic solutions by means of pulse radiolysis and electron paramagnetic resonance spectroscopy.^68^ The reactions with HO˙ were performed in acidic aqueous solution (pH = 5–6). It was found that THB–OH and HO˙ first undergo a RAF pathway which yields the radical adduct [HO–THB–OH]˙ with a rate constant larger than that of diffusion (≥1 × 10^10^ M^−1^ s^−1^). Then, the adduct suffers successive elimination of hydroxide anion (slower, rate constant = 2 × 10^5^ M^−1^ s^−1^) and proton (fast), thus yielding the phenoxyl radical THB–O˙. Although our investigation simulates the reactions of THB–OH and HO˙ in basic solution, some comparison of the results can be made. At pH = 5–6 THB–OH exists as a neutral form. According to our investigation, only the reactions (3) and (4) would occur in such conditions. The *k*_overall_ value of 3.25 × 10^8^ M^−1^ s^−1^ would be around 30 times smaller than that proposed by Wang *et al.*^68^ The two sets of results agree that the only products of the reactions of THB–OH and HO˙ are [HO–THB–OH]˙ and THB–O˙, where [HO–THB–OH]˙ is formed in an elementary reaction. Certainly, a two-step dehydration of [HO–THB–OH]˙ was not considered in our work. As hydrogen abstraction is a ubiquitous chemical reaction,^69^ we assume that HAT pathway can contribute to the formation of THB–O˙, independently of media acidity.

## 4. Conclusions

In this work, the scavenging capacity of two structurally similar compounds: THB–OH and THP–OH towards HO˙ was examined through investigation of the antioxidative mechanisms in two solvents of different polarity: benzene and water.

In benzene solution, both compounds conform to the HAT and RAF mechanisms. In water solution, the antioxidative mechanisms of the investigated compounds are far more complex, especially those of THB–OH. At physiological conditions, THB–OH exists in neutral and monoanionic forms, whereas THP–OH exists in the form of monoanion.

THB–OH and HO˙ undergo all four investigated mechanisms: HAT, RAF, SPLET, and even SET-PT. The behaviour of THB–O^−^ turned out to be quite intriguing. Namely, in the presence of the HO˙ radical, THB–O^−^ spontaneously transforms into THB–O˙, implying that HAT and RAF mechanisms should be examined as the reactions of THB–O˙ with HO˙. In the barrierless HAT reaction between THB–O˙ and HO˙ a diradical product is formed. This finding is surprising considering that diradicals are very rarely more stable than singlet molecules. In the case of the RAF mechanism, the two-state reactivity phenomenon was observed, indicating that the transformation of triplet reactants into singlet products takes place on two potential energy surfaces.

In the case of the reaction between THP–OH and HO˙ the operative antioxidative mechanisms are HAT, RAF and SPLET. In the case of the electron transfer reaction, the inverted-region phenomenon was observed. As a consequence, this particular electron transfer reaction is not diffusion controlled.

Despite pronounced differences in the operative reaction pathways in polar and nonpolar solvents, THB–OH and THP–OH are better scavengers of HO˙ in comparison to Tx. It should be pointed out as a final remark that the presence of the nitrogen atom in the ring decreases scavenging capacity towards HO˙.

## Conflicts of interest

The authors declare no competing financial interest.

## Supplementary Material

RA-010-D0RA08377A-s001
