# Supplementary material for: Comparison of the scavenging capacities of phloroglucinol and 2,4,6-trihydroxypyridine towards HO˙ radical: a computational study
Source: RSC Adv. 2020 Nov 27;10(71):43262–72. doi: 10.1039/d0ra08377a (PMC9058218; doi:10.1039/d0ra08377a)
Supplement: RA-010-D0RA08377A-s001 [file RA-010-D0RA08377A-s001.pdf]

Electronic Supplementary Material (ESI) for RSC Advances.
This journal is © The Royal Society of Chemistry 2020

RSC Advances

Supplementary information

associated with the paper

Comparison of the scavenging capacities of phloroglucinol and 2,4,6-trihydroxypyridine towards HO$^{\bullet}$ radical: a computational study

Žiko Milanović [a], Jelena Tošović [a,], Svetlana Marković [a], Zoran Marković [b]*

[a] University of Kragujevac, Faculty of Science, Department of Chemistry, 12 Radoja Domanovića, 34000 Kragujevac, Serbia

[b] University of Kragujevac, Institute for Information Technologies, Department of Science, Jovana Cvijića bb, 34000 Kragujevac, Serbia

*Corresponding author:

Phone/Fax: +381-34-336-223 /+381-34-335-039

E-mail: jelena.tosovic@pmf.kg.ac.rs

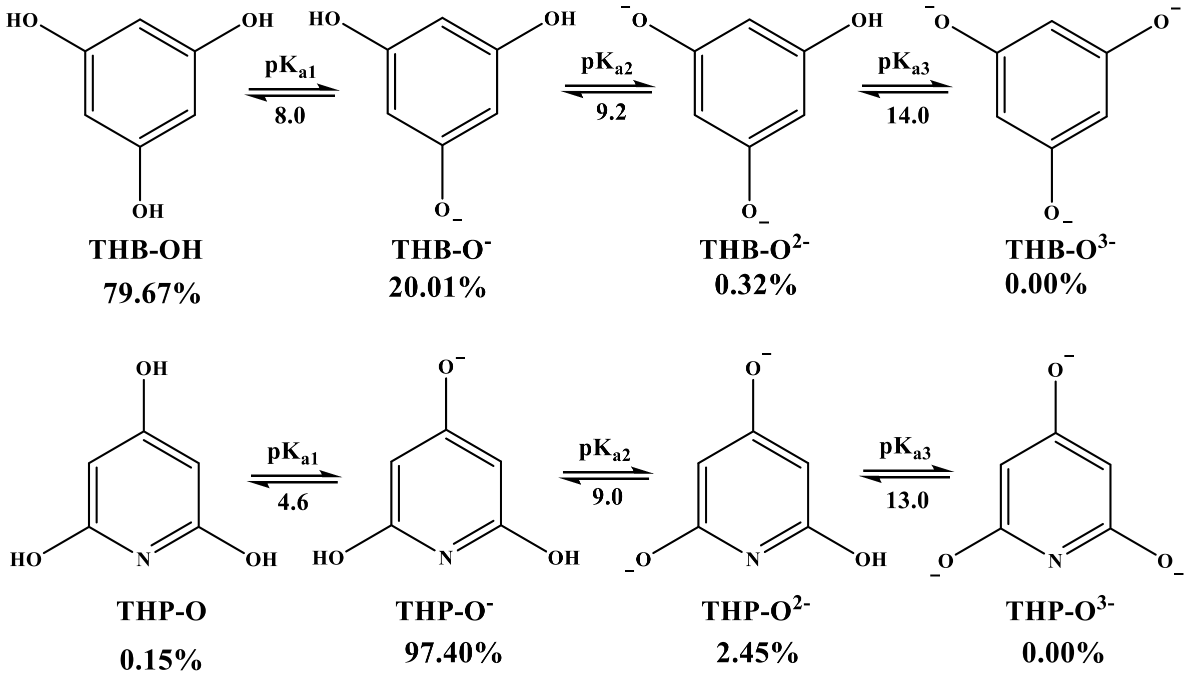

Fig. S1 Deprotonation sequences, pK_a values, and molar fractions of the investigated compounds at physiological conditions.

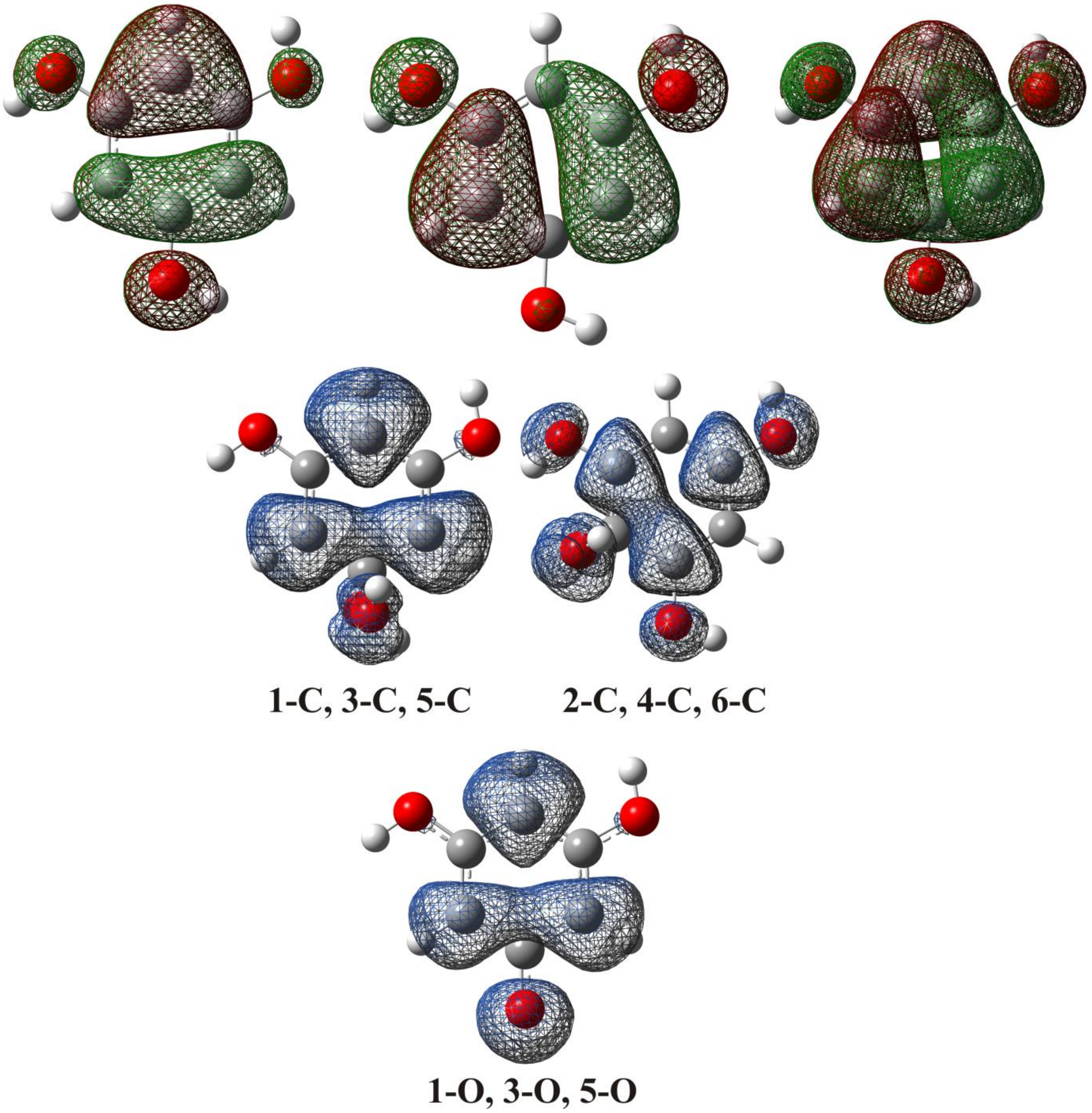

Fig. S2 Two degenerate HOMOs of **THB-OH** and their superposition (top), spin density surfaces of the **HO**$^{-}$**THBO**$^{\bullet}$ radicals formed in the RAF pathways (middle), spin density surface of the **THB-O**$^{\bullet}$ radical formed in the HAT pathway (bottom). All surfaces refer to benzene solution.

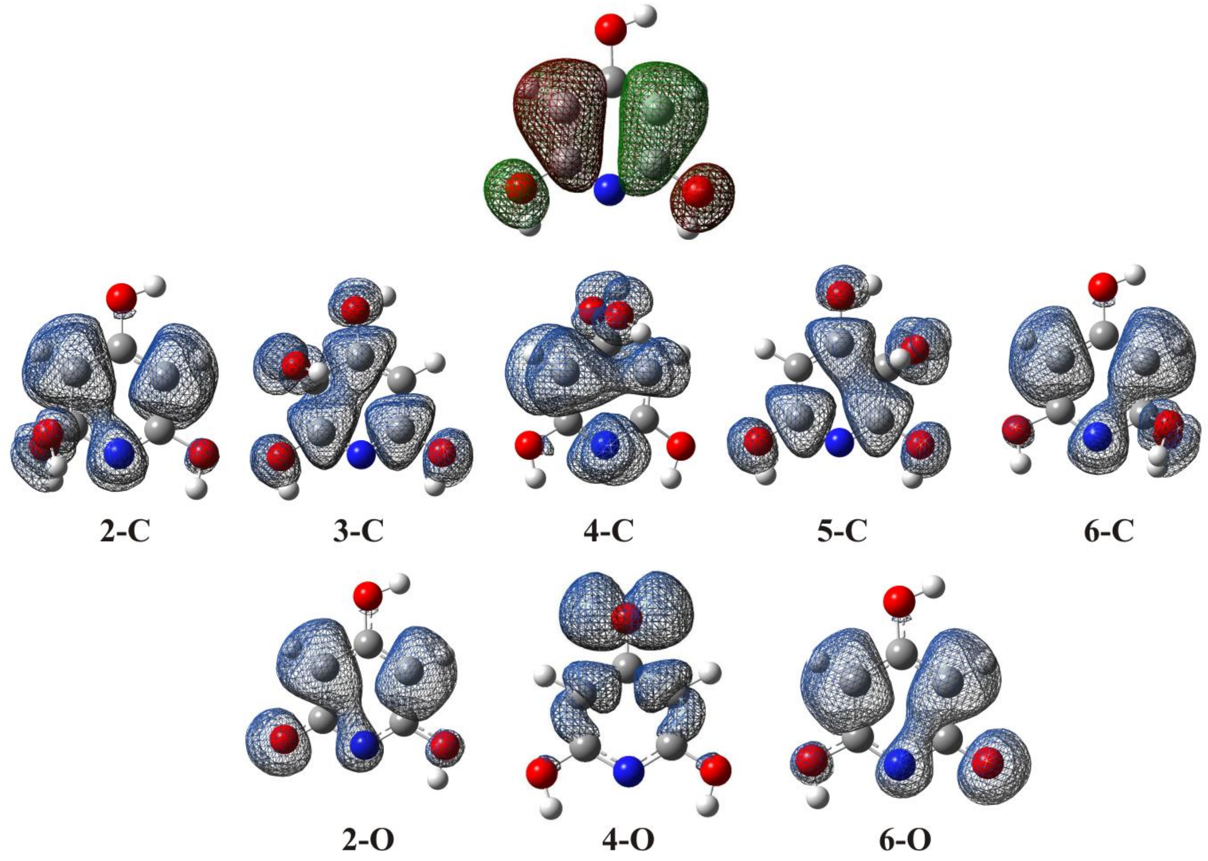

Fig. S3 HOMO of **THP-OH** (top), spin density surfaces of the **HO-THPO•** radicals formed in the RAF pathways (middle), spin density surfaces of the **THP-O•** radicals formed in the HAT pathway (bottom). All surfaces refer to benzene solution.

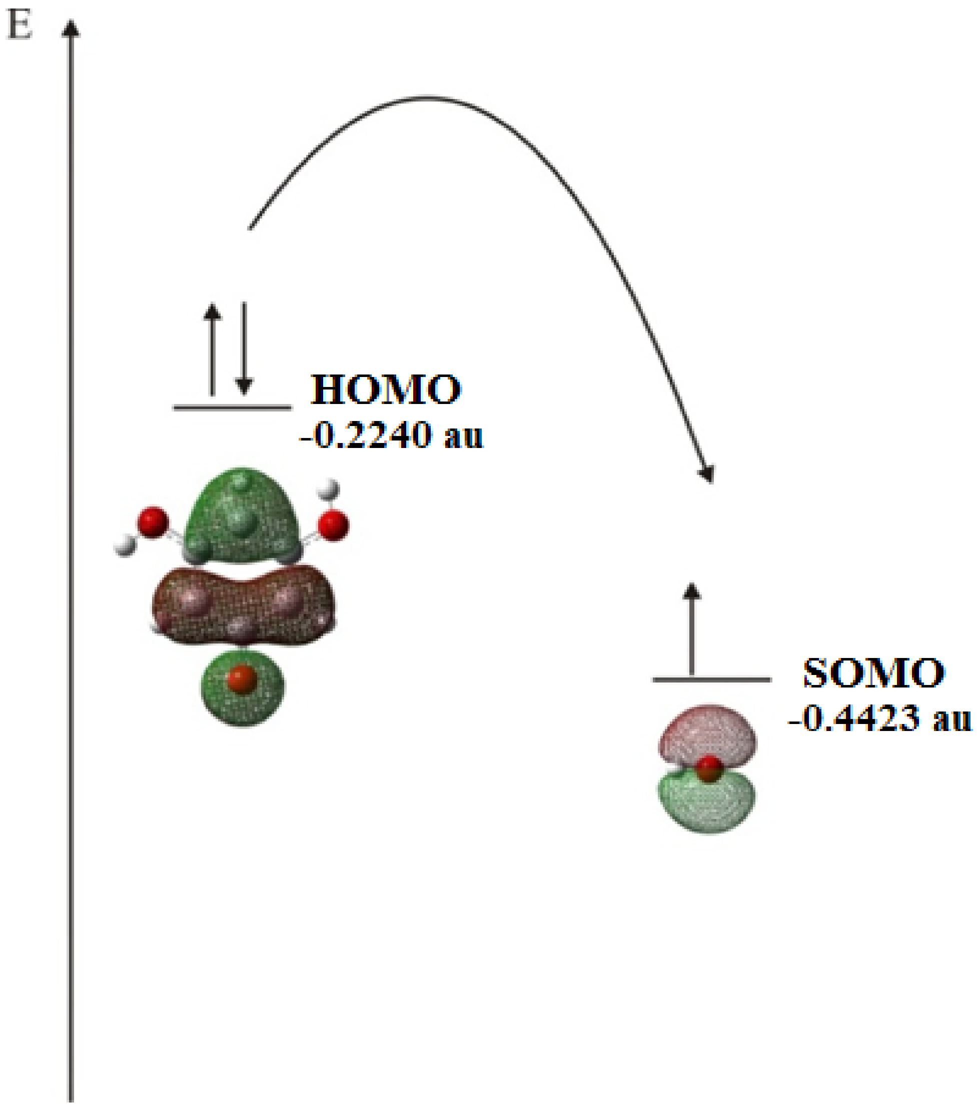

Fig. S4 Spontaneous electron transfer from the HOMO of the **THB-O⁻** monoanion to the SOMO of HO•.

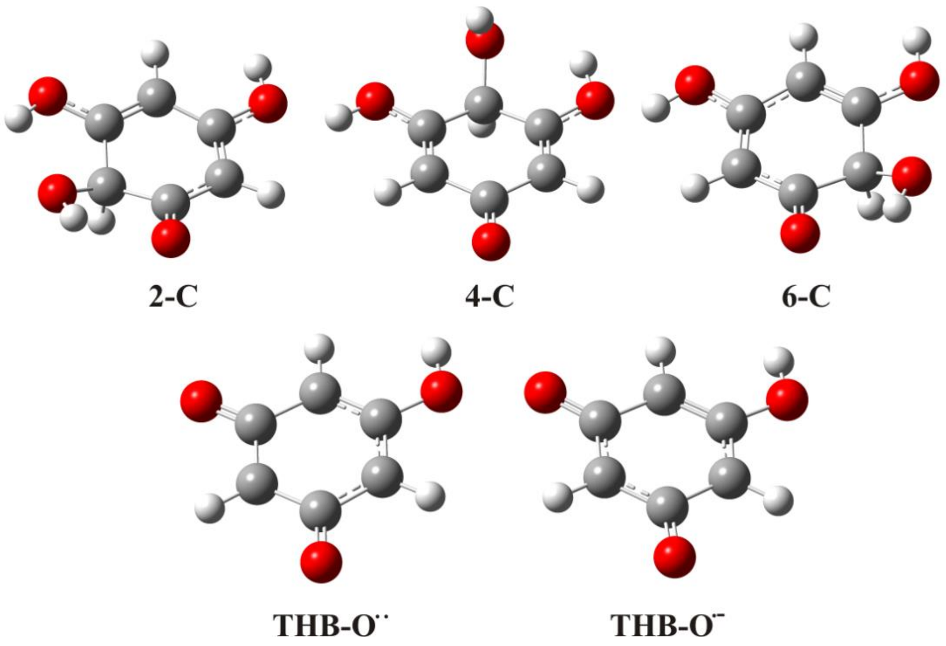

Fig. S5 Optimized geometries of intermediates and products of the reactions of **THB-O^{-}** with HO$^{\bullet}$ in the aqueous solution formed in: RAF pathways in different positions (**HO-THBO**, top), HAT pathway (**THB-O$^{\bullet\bullet}$**) and PT pathway (**THB-O$^{\bullet-}$**).

(16)

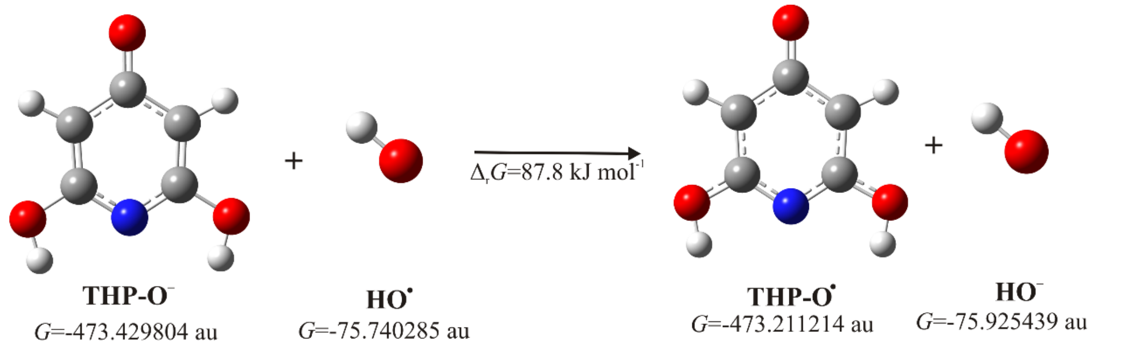

(17)

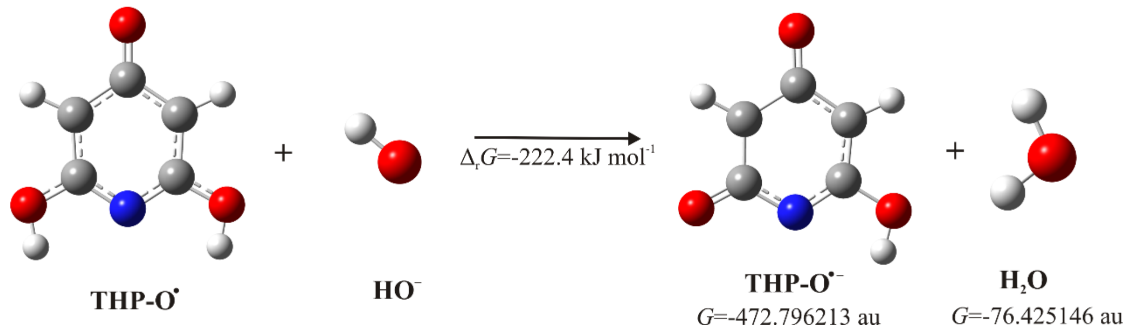

Fig. S6 The reactions of electron transfer (16) between **THP-O$^-$** and **HO$^\bullet$**, and proton transfer (17) between **THP-O$^\bullet$** and **HO$^-$** with corresponding Gibbs free energies (au) and reaction free energies (kJ mol^{-1}).

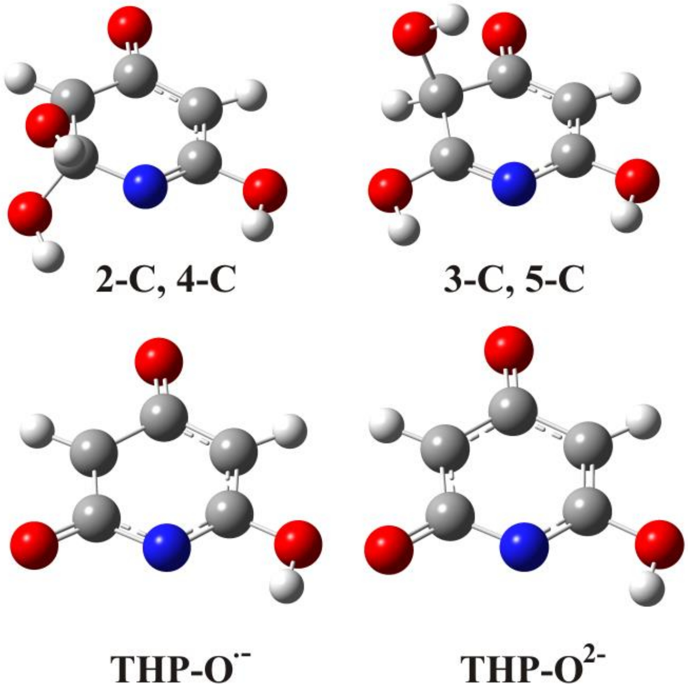

Fig. S7 Optimized geometries of intermediates and products of the reactions of **THP-O^{-}** with HO$^{\bullet}$ in the aqueous solution formed in: RAF pathways in different positions (**[HO-THP-O]$^{\bullet-}$**, top), HAT pathway (**THP-O$^{\bullet-}$**) and SPL pathway (**THP-O^{2-}**).

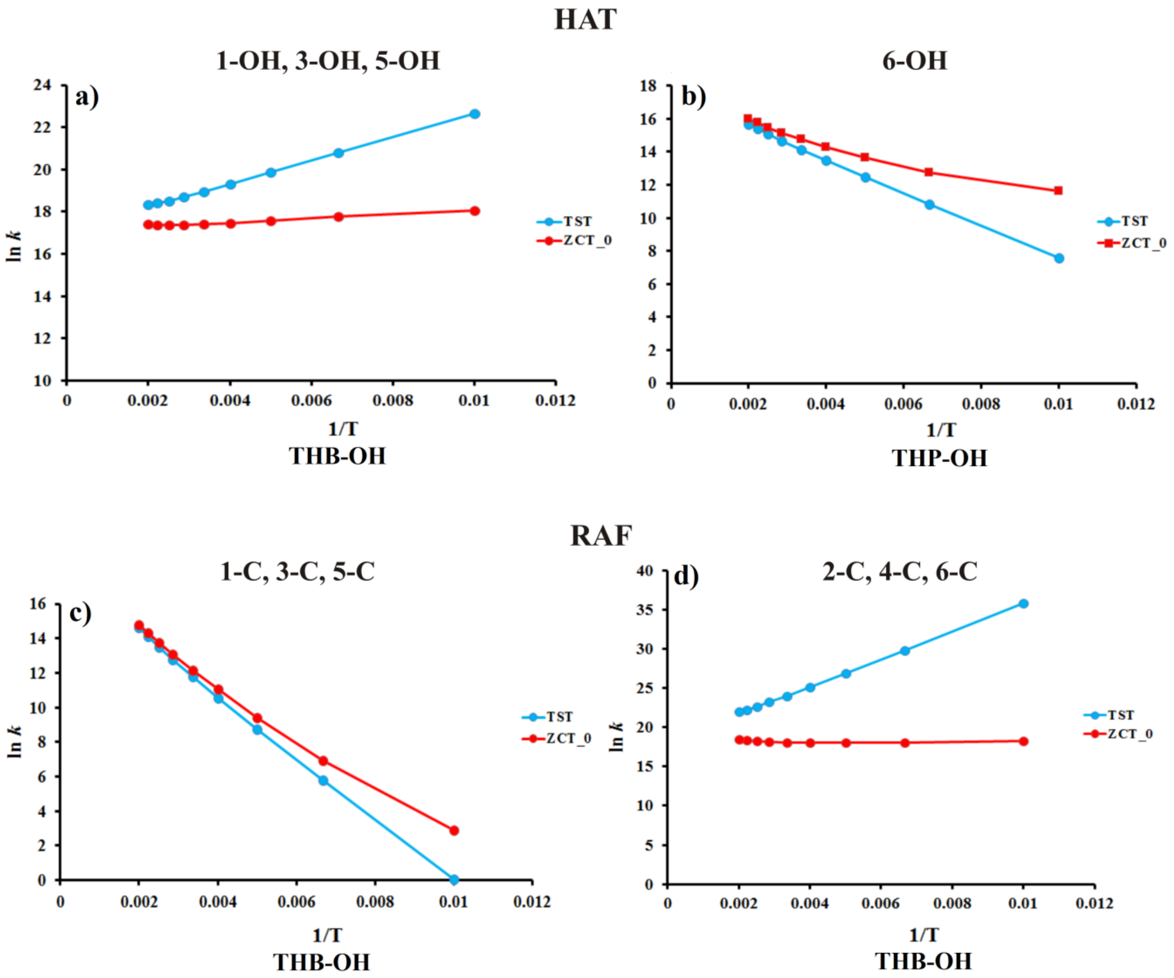

Fig. S8 Dependence of ln$k_{ZCT\text{-}0}$ (red) and lnk_{TST} (blue) on reciprocal temperature for the reactions of $HO^{\bullet}$ with: a) **THB-OH** in the 1-OH, 3-OH, and 5-OH positions (HAT), b) **THP-OH** in the 6-OH position (HAT), c) **THB-OH** in the 1-C, 3-C, and 5-C positions (RAF), and d) **THB-OH** in the 2-C, 4-C, and 6-C positions (RAF), respectively. All data refer to the benzene solution.

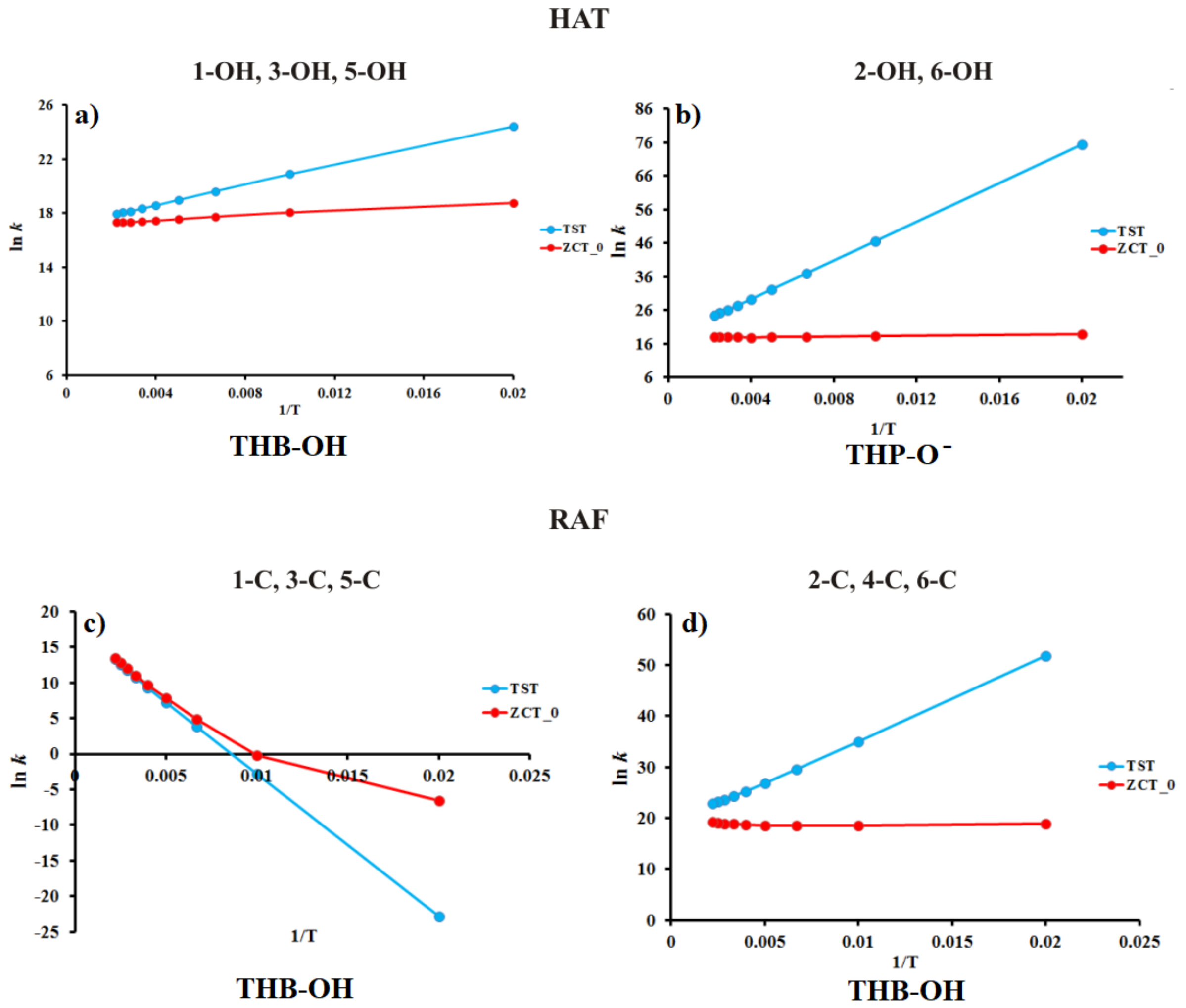

Fig. S9 Dependence of ln$k_{ZCT\text{-}0}$ (red) and lnk_{TST} (blue) on reciprocal temperature for the reactions of $HO^{\bullet}$ with: a) **THB-OH** in the 1-OH, 3-OH, and 5-OH positions (HAT), b) **THP-O^{-}** in the 2-OH and 6-OH positions (HAT), c) **THB-OH** in the 1-C, 3-C, and 5-C positions (RAF) and d) **THB-OH** in the 2-C, 4-C, and 6-C positions (RAF), respectively. All data refer to the water solution.

Comment on Figs. S8 and S9:

Figs. S8 and S9 illustrate the dependence of k_{TST} and $k_{ZCT\text{-}0}$ on temperature. All presented graphs show that the difference between the two rate constants is pronounced at lower temperatures but rapidly decreases with increasing temperature. In some cases (the HAT pathways of **THB-OH** in both solvents and that of **THP-O^{-}** in water solution, as well as the RAF reactions of **THB-OH** in the positions 2-C, 4-C, and 6-C in both solvents) the k_{TST} values decrease with increasing temperature, and the $k_{ZCT\text{-}0}$ values are significantly smaller at all temperatures. It is evident that the conventional TST is not adequate for assessing the rates of such HAT and RAF reaction pathways. Bearing in mind that these paths are characterized with extremely small activation energy, the failure of TST can be attributed to the flat potential energy surface.

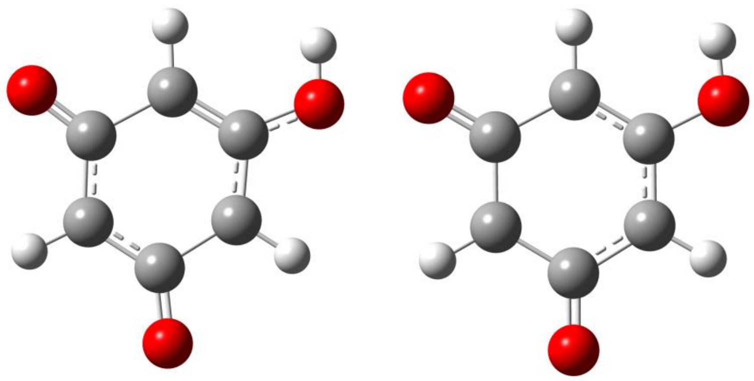

a) b)

Fig. S10 Optimized structures of a) **THB=O** (singlet state) and b) **THB-O$^{\bullet\bullet}$** (triplet state) in water solution.

Table S1 Geometrical parameters of a) **THB=O** (singlet state) and b) **THB-O$^{\bullet\bullet}$** (triplet state) in water solution.

**Bond lengths (pm)**	**a)**	**b)**	**Bond angles (º)**	**a)**	**b)**
C1-C2	148.8	145.0	O1-C1-C2	128.8	120.8
C2-C3	142.3	145.0	C1-C2-C3	120.8	121.2
C3-C4	151.6	145.0	C2-C3-O3	125.4	120.8
C4-C5	136.2	138.8	C2-C3-C4	120.1	117.5
C5-C6	140.7	139.7	O3-C3-C4	114.6	121.7
C1-O1	124.8	124.1	C3-C4-C5	120.8	121.3
C3-O3	122.8	123.9	C4-C5-O5	126.9	122.5
C5-O5	134.4	134.7	C5-C6-C1	126.6	121.3
			C6-C1-O1	115.9	121.7

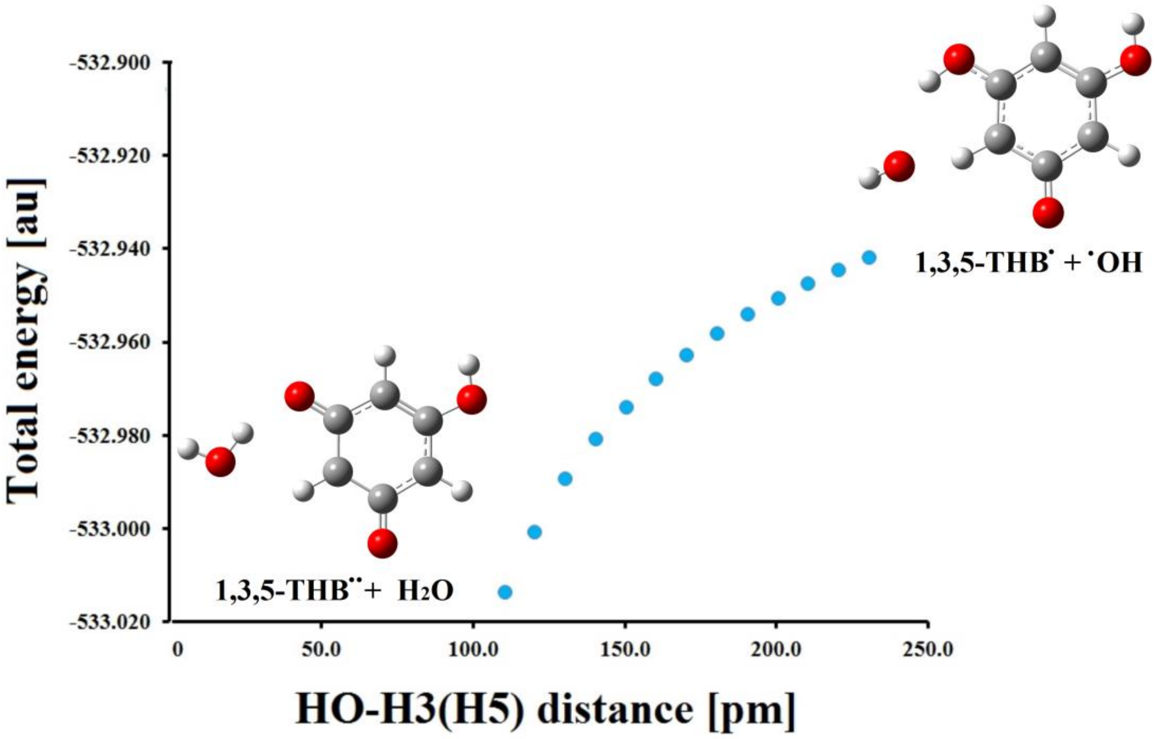

Fig. S11 Dependence of total energy on the HO–H3(H5) distance in the HAT pathway of **THB-O•** with HO•.

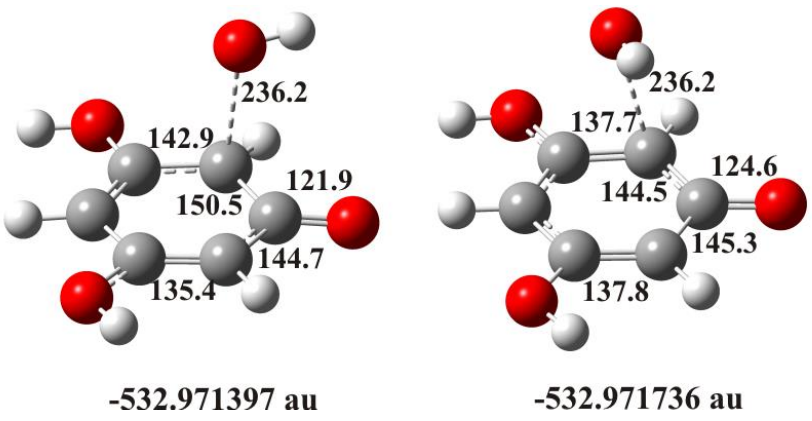

Fig. S12 Structure of the spin crossing point in the RAF C-6 pathway of **THB-O•** with HO• in the singlet (left) and triplet (right) states.

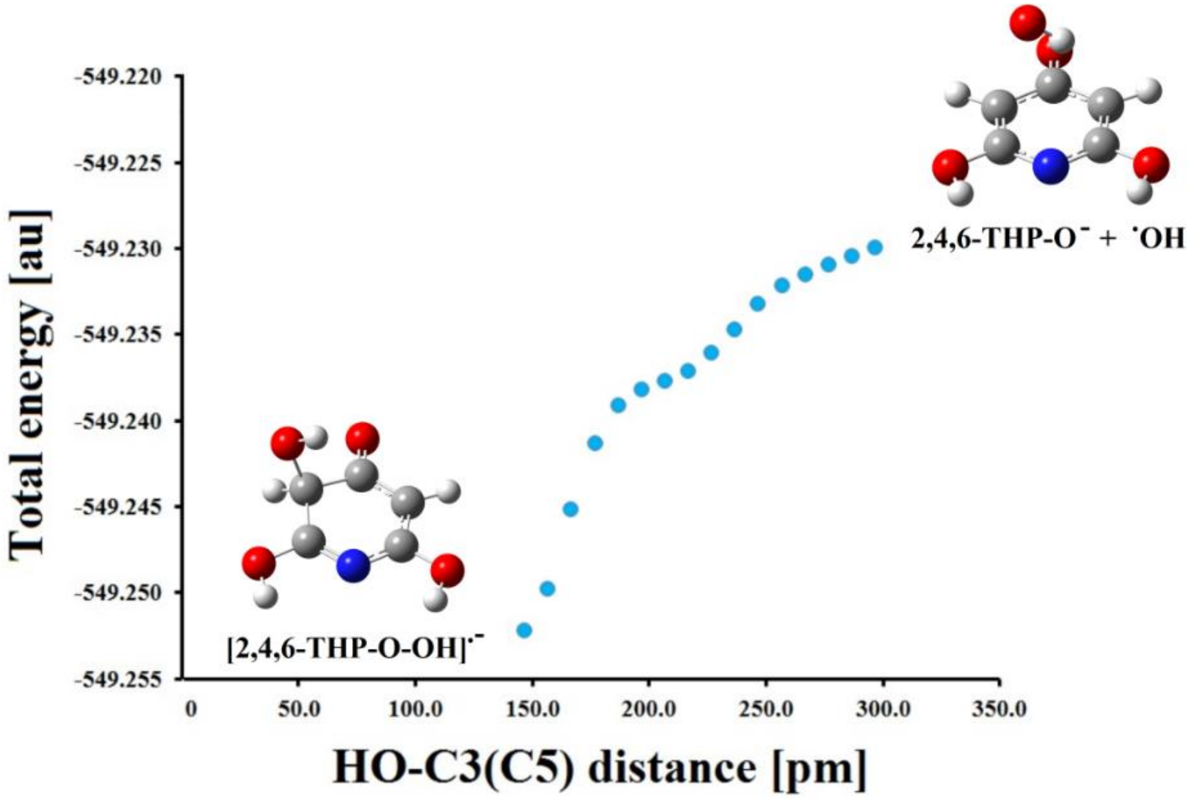

Fig. S13 Dependence of total energy on the HO–C3(C5) distance in the RAF pathway of **THP-O**$^{-}$ and HO$^{\bullet}$.

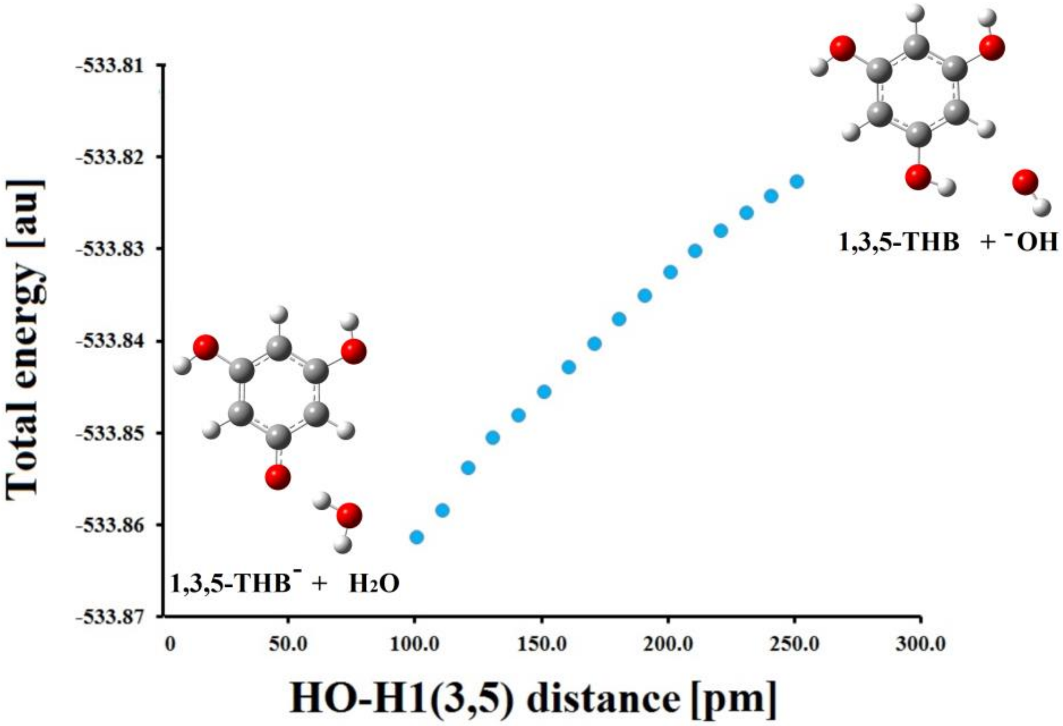

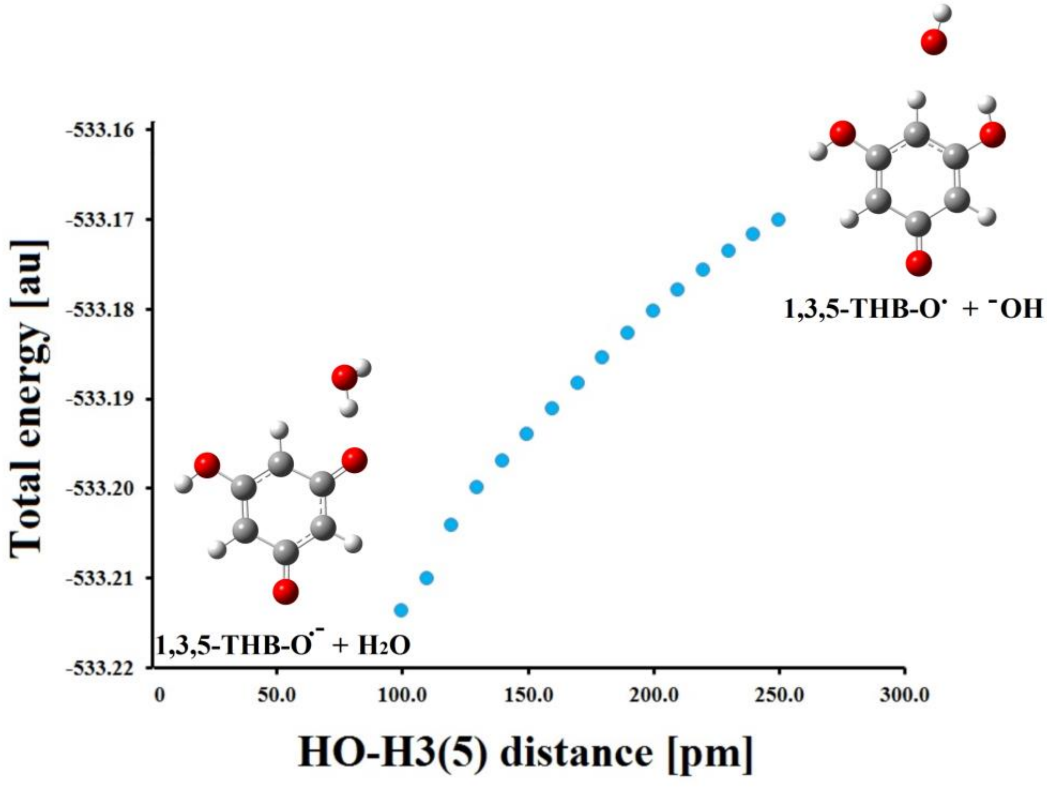

Fig. S14 Dependence of total energy on the HO–H1(3,5) distance in the proton transfer reaction from **THB-OH** to HO^- (top), and on the HO– H3(5) distance in the proton transfer reaction from **THB-O•** to HO^- (bottom).

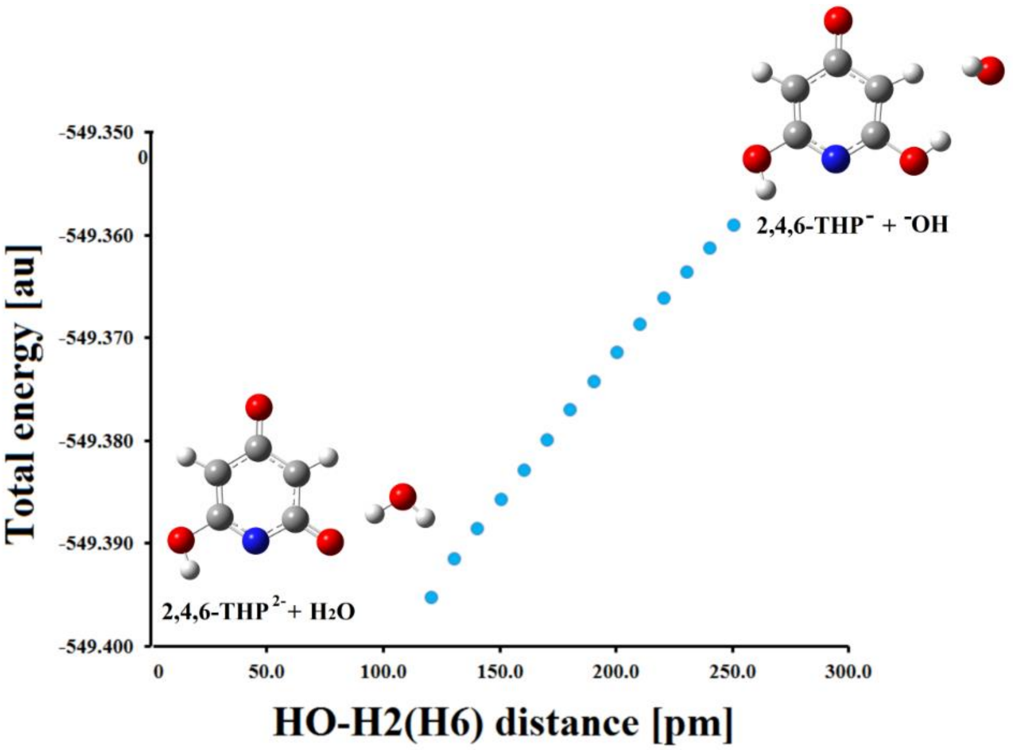

Fig. S15 Dependence of total energy on the HO– H2(H6) distance in the proton transfer reaction from **THP-O⁻** to HO⁻.

Table S2 Branching ratios (*Γ*) in % for all favorable antioxidative pathways.

Compound	*Mechanism*	**BENZENE (ε = 2.2706)**		**WATER (ε =78.3553)**	
		Position	*Γi*	*Position*	*Γi*
**THB-OH**	***HAT***	**1-OH**	11.20	**1-OH**	0.36
		**3-OH**	11.20	**3-OH**	0.36
		**5-OH**	11.20	**5-OH**	0.36
	RAF	**1-C**	0.10	**1-C**	0.00
		**2-C**	22.00	**2-C**	1.57
		**3-C**	0.10	**3-C**	0.00
		**4-C**	22.00	**4-C**	1.57
		**5-C**	0.10	**5-C**	0.00
		**6-C**	22.00	**6-C**	1.57
**THB-O$^-$**	***SPLET***	/	/	**SPL**	19.60
		/	/	**ET**	19.67
	SET-PT	/	/	**SET**	
		/	/	**PT**	4.92
	SPLET	/	/	**SPL**	
		/	/	**ET**	20.47
	HAT	/	/	**3-OH**	4.92
		/	/	**5-OH**	4.92
	RAF	/	/	**1-C**	4.92
		/	/	**2-C**	4.92
		/	/	**4-C**	4.92
		/	/	**6-C**	4.92
**THP-OH**	***HAT***	**2-OH**	0.40	/	/
		**4-OH**	6.30		
		**6-OH**	1.11	/	/
	RAF	**2-C**	0.00	/	/
		**3-C**	43.40	/	/
		**5-C**	48.80	/	/
		**6-C**	0.00	/	/
**THP-O$^-$**	***HAT***	/	/	**2-OH**	0.64
		/	/		
		/	/	**6-OH**	0.64
	RAF	/	/	**2-C**	0.50
		/	/	**3-C**	32.46
		/	/	**5-C**	32.46
		/	/	**6-C**	0.50
	SPLET	/	/	**SPL**	32.46
		/	/	**ET**	0.35

Cartesian coordinates of equilibrium geometries (benzene)

THP-OH

G = -473.878456 au

0 1	x	y	z
C	-0.59354500	1.17466100	-0.00000400
C	0.79477800	1.14733600	-0.00002000
C	1.39553500	-0.11003400	-0.00009700
C	0.61761600	-1.26352100	-0.00000200
C	-0.75801300	-1.08620500	0.00006600
N	-1.36794400	0.09587100	0.00001300
H	1.36376900	2.06740200	-0.00001000
H	1.05957600	-2.24870900	0.00000800
O	2.73539900	-0.26695600	0.00002500
H	3.17230900	0.59067100	0.00016100
O	-1.20842400	2.37254000	0.00001100
H	-2.16037900	2.21238900	-0.00002600
O	-1.54402300	-2.17897600	-0.00001200
H	-2.46150800	-1.87913100	-0.00006900

THP-O• (2-OH)

G = -473.232110 au

0 2	x	y	z
C	0.95110800	-0.89775300	0.00005600
C	-0.43991100	-1.26162300	-0.00004400
C	-1.36932000	-0.24292900	-0.00000800
C	-0.90976100	1.07410200	-0.00004000
C	0.52087800	1.35217600	-0.00039100
N	1.42934200	0.30157000	-0.00000400
H	-0.71100900	-2.31004000	-0.00002300
H	-1.59636500	1.90991000	0.00011700
O	-2.70193500	-0.43905900	0.00006100
H	-2.91160900	-1.37912100	0.00010500
O	1.79048900	-1.93849700	0.00002000
H	2.69215000	-1.58755200	0.00003800
O	0.91188100	2.51655300	0.00021300

THP-O• (4-OH)

G = -473.201194 au

0 2	x	y	z
C	-0.66450800	-1.11204700	-0.00000300
C	0.75429900	-1.19028300	-0.00000600
C	1.56328900	-0.00003600	-0.00000300
C	0.75425700	1.19029000	-0.00000200

C	-0.66438100	1.11215200	0.00000700
N	-1.39451800	-0.00002400	0.00000300
H	1.21632300	-2.16935700	-0.00001100
H	1.21668200	2.16915800	-0.00000800
O	2.80040800	0.00006100	0.00000500
O	-1.31257800	-2.25881600	0.00000100
H	-2.26493500	-2.07755300	0.00000500
O	-1.31271900	2.25876000	-0.00000100
H	-2.26507100	2.07742400	-0.00000100

THP-O• (6-OH)

G = -473.232636 au

0 2	x	y	z
C	-0.38869700	1.38665800	0.00012300
C	1.01004600	0.97139500	0.00000300
C	1.33917100	-0.37481700	-0.00002100
C	0.31055800	-1.30428100	0.00002400
C	-1.03772600	-0.81134000	0.00007100
N	-1.39557200	0.43030300	0.00003700
H	1.75809200	1.75497600	-0.00003900
H	0.50132400	-2.36859800	0.00000700
O	2.60466800	-0.83952600	0.00000500
H	3.23580800	-0.11209600	0.00006200
O	-0.66519900	2.58289300	-0.00009700
O	-1.97542600	-1.76398400	-0.00007900
H	-2.83866700	-1.32715000	-0.00012000

THP-O⁻ (2-OH)

G = -473.382725 au

-1 1	x	y	z
C	0.85817200	-0.96464700	-0.00004100
C	-0.49856600	-1.27266700	0.00007000
C	-1.35418100	-0.16016700	0.00002200
C	-0.85957500	1.12012200	0.00007400
C	0.56535700	1.34521100	0.00051000
N	1.39489300	0.23414400	0.00005600
H	-0.85063000	-2.29559800	0.00003500
H	-1.52652900	1.97199700	-0.00015700
O	-2.71539900	-0.33376500	-0.00005000
H	-2.90446900	-1.27608500	-0.00000100
O	1.73785800	-2.01522500	-0.00011100
H	2.61135100	-1.60488800	-0.00014400
O	1.05738900	2.49379700	-0.00033100

THP-O^{-} (4-OH)

G = -473.389300 au

-1 1	x	y	z
C	-0.61758400	-1.12961100	-0.00000700
C	0.75255100	-1.21007800	0.00001300
C	1.53477600	0.00140400	0.00013300
C	0.74978200	1.21163600	0.00000300
C	-0.61991300	1.12868000	-0.00003700
N	-1.33906300	-0.00146200	-0.00000200
H	1.24684800	-2.17271800	-0.00003600
H	1.24308300	2.17477100	-0.00004200
O	2.79171700	0.00299100	-0.00005200
O	-1.35309200	-2.28417000	-0.00001400
H	-2.27590700	-2.00604300	0.00000800
O	-1.35835400	2.28134000	-0.00000500
H	-2.28042400	2.00074800	0.00002600

THP-O^{-} (6-OH)

G = -473.384238 au

-1 1	x	y	z
C	-0.35794700	1.40248500	0.00013100
C	1.01474900	0.95702200	0.00002900
C	1.30619400	-0.38748200	0.00001400
C	0.29213600	-1.35175600	0.00002200
C	-0.99870900	-0.83581800	-0.00001500
N	-1.34595200	0.43384700	0.00000400
H	1.79865800	1.70653700	-0.00001900
H	0.49504000	-2.41205300	0.00001700
O	2.59730700	-0.84974100	0.00001500
H	3.18918600	-0.09166600	0.00004000
O	-0.66120500	2.61510900	-0.00009500
O	-2.03218900	-1.73457300	-0.00005400
H	-2.83106900	-1.19281100	-0.00007200

[HO-THP-OH]$^{\bullet}$ (2-OH)

G = -549.628538 au

0 2	x	y	z
C	-0.27176100	1.40057200	0.00009500
C	-1.48237300	0.61478000	0.00011800
C	-1.35777800	-0.76220100	0.00016000
C	-0.09750800	-1.33439900	-0.00007200
C	1.13094400	-0.47263000	0.00012100
N	0.92674100	0.95576500	0.00028600
H	-2.43786000	1.12241700	0.00055300

H	0.03800600	-2.40749200	-0.00079100
O	-2.42185800	-1.60015000	0.00006200
H	-3.24396000	-1.09956900	-0.00145000
O	-0.48789500	2.72870200	-0.00000900
H	0.37404400	3.16546200	0.00016100
O	1.85318500	-0.81388300	-1.15768500
H	2.43862800	-0.07332400	-1.35690800
O	1.85346200	-0.81463700	1.15726500
H	2.43965400	-0.07483700	1.35684700

[HO-THP-OH]$^{\bullet}$ (3-OH)

G = -549.626702 au

0 2	x	y	z
C	-1.54659200	-0.33234100	0.07369200
C	-0.63891400	-1.40334100	-0.04354300
C	0.68291900	-1.11897500	-0.22608200
C	1.21194400	0.27422600	-0.14478000
C	0.06487300	1.23495800	-0.23124600
N	-1.19553600	0.95316100	-0.05593300
H	-1.01088400	-2.42067600	-0.02137300
H	1.92727800	0.46556800	-0.95046500
O	1.64990500	-2.03936300	-0.43079600
H	1.27185200	-2.92510100	-0.45183800
O	-2.84962400	-0.61042700	0.29017300
H	-3.32852200	0.22710900	0.30774800
O	0.43612500	2.50447800	-0.45800200
H	-0.35914500	3.05337600	-0.46821400
O	1.98777300	0.48476700	1.04849400
H	1.42935300	0.28478900	1.80846700

[HO-THP-OH]$^{\bullet}$ (4-OH)

G = -549.615301 au

0 2	x	y	z
C	0.85008400	1.18600400	-0.01659900
C	-0.49166600	1.23323000	-0.02774700
C	-1.29350700	-0.02967700	-0.01790300
C	-0.41462500	-1.24327600	-0.04265500
C	0.99133600	-1.10699800	-0.00828600
N	1.61872300	0.03144000	0.00398100
H	-1.01572100	2.18046000	-0.04690400
H	-0.88283200	-2.21788900	-0.06997900
O	-2.15229200	-0.15410100	-1.12074800
H	-2.76743400	0.58863600	-1.10251000
O	1.58393400	2.32053300	-0.02221700

H	2.51415300	2.06643400	-0.01817500
O	1.70170100	-2.24722500	0.00731000
H	2.63929900	-2.01394800	0.01655500
O	-2.13260000	-0.09347600	1.13626500
H	-1.67420600	0.33468700	1.86740400

[HO-THP-OH]• (5-OH)

G = -549.628487 au

0 2	x	y	z
C	-0.06403400	1.22925400	-0.15496500
C	-1.16862900	0.22613700	-0.27436800
C	-0.60357800	-1.15400500	-0.17119900
C	0.72433000	-1.40048300	-0.02044200
C	1.60443400	-0.30122800	0.05169400
N	1.20507600	0.97408000	-0.00620200
H	-1.69367800	0.36243700	-1.23177900
H	1.10228300	-2.41102400	0.05644700
O	-1.49760900	-2.16334200	-0.25344600
H	-2.38114100	-1.80793300	-0.09210800
O	-0.48119500	2.50273400	-0.25539700
H	0.29262200	3.07726100	-0.18691300
O	2.92280100	-0.54027800	0.21126800
H	3.37430700	0.31161200	0.24801100
O	-2.22493500	0.40870700	0.68470200
H	-1.83755100	0.38847100	1.56842100

[HO-THP-OH]• (6-OH)

G = -549.628584 au

0 2	x	y	z
C	-1.10175500	-0.51625200	0.00002200
C	0.16523800	-1.32223500	0.00065300
C	1.39004500	-0.70017800	0.00007000
C	1.45423400	0.68955000	-0.00030200
C	0.21292200	1.42137000	0.00011100
N	-0.96430800	0.91924200	0.00040700
H	0.05522500	-2.40018000	0.00129200
H	2.39592400	1.21925000	-0.00077600
O	2.56817100	-1.37145200	0.00015700
H	2.41027600	-2.32092400	0.00006300
O	-1.80737700	-0.89037100	-1.15780700
H	-2.43057600	-0.18071500	-1.35484800
O	0.36590100	2.75770500	0.00018700
H	-0.51490600	3.15479700	0.00039300
O	-1.80899600	-0.89085500	1.15667900

H	-2.43148700	-0.18067400	1.35397600

THB-OH

G = -457.800070 au

0 1	x	y	z
C	-1.06921500	0.90587400	0.00000700
C	0.24375100	1.35957300	0.00000100
C	1.31906700	0.47288500	0.00000700
C	1.05576800	-0.89111100	0.00000400
C	-0.25008700	-1.37905300	-0.00000800
C	-1.29944000	-0.46887500	0.00000000
H	-1.90898600	1.59024000	0.00001500
H	2.33161800	0.85846900	0.00001200
H	-0.42292800	-2.44852600	-0.00003100
O	2.05211700	-1.81645000	0.00001000
H	2.90732600	-1.37609900	-0.00008200
O	0.54752100	2.68497200	-0.00000300
H	-0.26093700	3.20638100	-0.00002300
O	-2.59928100	-0.86817700	-0.00000800
H	-2.64801000	-1.82898900	0.00005300

THB-O$^{•}$ (1-OH, 3-OH, 5-OH)

G = -473.232110 au

0 2	x	y	z
C	1.16758400	0.86382500	-0.00000900
C	-0.14139400	1.48320400	-0.00000200
C	-1.31001200	0.62453400	-0.00000100
C	-1.15453400	-0.73357600	0.00000300
C	0.13520300	-1.31466400	0.00000700
C	1.28301600	-0.50579000	-0.00000300
H	2.04336500	1.49986600	-0.00001600
H	-2.28533400	1.09701600	-0.00000400
H	0.21402400	-2.39655300	0.00001900
O	-2.18165300	-1.61832700	0.00000300
H	-3.02334500	-1.15091800	0.00006500
O	-0.25859000	2.72205800	-0.00000200
O	2.52687600	-1.04706800	-0.00000100
H	2.47903700	-2.00790800	-0.00003700

THB-O$^{–}$ (1-OH, 3-OH, 5-OH)

G = -473.232110 au

-1 1	x	y	z
C	1.05791600	0.96526700	-0.00000800
C	-0.28176400	1.48583600	-0.00000300

C	-1.32584200	0.49262800	0.00000200
C	-1.03667700	-0.85744600	0.00000700
C	0.27249400	-1.34520200	0.00000500
C	1.29834700	-0.39389400	-0.00000400
H	1.89123600	1.65818400	-0.00001600
H	-2.35658300	0.83717600	0.00000000
H	0.46631600	-2.41118600	0.00001500
O	-2.04051000	-1.80450200	0.00001100
H	-2.88374200	-1.34322100	0.00004900
O	-0.53271000	2.72765100	-0.00000600
O	2.61602700	-0.80541500	-0.00000600
H	2.63347100	-1.76595400	-0.00003400

[HO-THB-OH]• (1-OH, 3-OH, 5-OH)

G = -533.548846 au

0 2	x	y	z
C	-0.43362300	-1.25309200	-0.02050100
C	-1.26514800	-0.00166600	-0.01617000
C	-0.45269200	1.25779900	-0.02232700
C	0.89919300	1.23278400	-0.00712200
C	1.62486000	0.00714800	-0.00096000
C	0.92613900	-1.22012000	-0.01284400
H	-0.96209500	-2.19994600	-0.02660400
H	-1.01569500	2.18438900	-0.03500500
H	2.70777700	0.04189000	0.00822900
O	1.68324800	2.34777400	0.00521400
H	1.13083300	3.13527400	-0.02933700
O	-2.11714600	0.05364000	-1.13513400
H	-2.74164600	-0.67830400	-1.06379700
O	1.60400600	-2.40450700	-0.01108700
H	2.55221100	-2.24565800	-0.02500600
O	-2.15005500	-0.02314000	1.10938700
H	-1.62418200	-0.16490900	1.90403000

[HO-THB-OH]• (2-OH, 4-OH, 6-OH)

G = -533.552767 au

0 2	x	y	z
C	-1.02269700	1.19708000	-0.00125600
C	0.33131200	1.24989900	-0.14851400
C	1.19506000	0.03712100	-0.26814700
C	0.38839900	-1.22031400	-0.17927800
C	-0.96624800	-1.25250000	-0.03376700
C	-1.68822800	-0.04562600	0.04780400
H	-1.61076600	2.10641100	0.06284400

H	1.74610100	0.06867700	-1.22054800
H	-1.47003700	-2.21075400	0.03340700
O	1.09955100	-2.37266900	-0.26846900
H	2.02186100	-2.17448800	-0.06077600
O	1.04612000	2.39949300	-0.25399900
H	0.46083700	3.16088300	-0.18436100
O	-3.03922000	-0.01261600	0.20682500
H	-3.39293000	-0.90719600	0.20903500
O	2.26262800	-0.00261400	0.70079800
H	1.86672100	0.06975600	1.57811100

Cartesian coordinates of transition states (benzene)

THP-O• (2-OH)

G = -549.597810 au

0 2	x	y	z
H	-2.26561100	-1.27501800	0.09089400
O	-1.47776800	-1.86726500	-0.36775100
C	-0.53114800	-0.98330000	-0.34645100
C	-0.92144600	0.39957500	-0.45970500
C	1.60982200	-0.40787900	0.09141400
C	0.05330400	1.37282900	-0.13183300
O	-2.72392600	-0.13908800	0.73134700
H	-2.42684800	-0.10059700	1.64951000
O	-0.33832400	2.65316900	-0.19252500
H	0.39923500	3.24513300	-0.00681400
O	2.87271100	-0.77745300	0.29373600
H	2.91477300	-1.74193500	0.22698500
H	-1.77872900	0.68779800	-1.04810900
C	1.33471700	0.97276000	0.17248600
N	0.72736900	-1.36589000	-0.14094700
H	2.13255300	1.66704000	0.40024600

THP-O• (4-OH)

G = -549.600153 au

0 2	x	y	z
H	-2.47962300	-0.91086300	0.14581400
O	-1.80650900	-1.66111700	-0.35700200
C	-0.72448700	-0.94687800	-0.35026800
C	0.55315000	-1.46166400	-0.15511400
C	-0.88660700	0.48451900	-0.45060100
C	1.57473200	-0.54955900	0.07884700
C	0.25409600	1.27939500	-0.15254600

O	-2.73819600	0.22958700	0.74622300
H	-2.43629700	0.20999300	1.66314600
H	0.73320500	-2.52411300	-0.08355000
N	1.44338300	0.79070600	0.11571300
O	0.09933700	2.59959700	-0.19234200
H	0.95502300	3.01325900	-0.01243300
O	2.79548300	-1.01930100	0.32721500
H	3.38019600	-0.26858600	0.49733900
H	-1.68241700	0.92036200	-1.03497100

THP-O• (6-OH)

G = -549.598800 au

0 2	x	y	z
H	2.19258700	-1.36779300	0.09858400
O	1.37613400	-1.93849200	-0.34350000
C	0.46705600	-1.01686600	-0.33673500
C	0.92475900	0.34709500	-0.46777900
C	-1.64034200	-0.32361300	0.08686900
C	0.00697600	1.37161300	-0.12916000
O	2.70285500	-0.24035500	0.71215900
H	2.41707000	-0.19217800	1.63338700
O	0.36647800	2.66325200	-0.14622700
H	1.31416000	2.74823500	-0.30102100
O	-2.92008600	-0.62746700	0.28262600
H	-3.01164400	-1.58897300	0.22228900
H	1.78337600	0.57085000	-1.08396800
C	-1.29355500	1.04013800	0.16711800
N	-0.80677100	-1.33187900	-0.13204700
H	-2.04056800	1.78731700	0.39271500

[HO-THP-OH]• (2-OH)

G = -549.594130 au

0 2	x	y	z
O	2.35532400	-0.50031300	-0.77146700
H	2.90323400	0.28545900	-0.65067300
C	1.13445600	-0.21829600	-0.30952400
C	-0.45272000	1.37990100	-0.03241500
C	0.16213400	-1.25260500	-0.36134200
C	-1.50496900	0.43873800	0.04036300
C	-1.17699000	-0.88775900	-0.15502500
H	-2.51935100	0.77651000	0.20431300
O	1.38153800	-0.82774500	1.51728500
H	0.60771700	-0.51294800	2.00709100
H	0.45702700	-2.27627300	-0.53694100

O	-2.08107100	-1.88530600	-0.15965400
H	-2.97109100	-1.53514200	-0.04503400
O	-0.79597000	2.66781100	0.10194100
H	0.01114200	3.19581200	0.03977700
N	0.80875500	1.09587600	-0.23081800

[HO-THP-OH]• (3-OH)

G = -549.604303 au

0 2	x	y	z
O	-0.44693000	-2.47822600	-0.56468400
H	0.34021200	-3.02647400	-0.44905800
C	-0.09710300	-1.20296400	-0.36570500
C	1.49563900	0.33747000	0.07546300
C	-1.12342100	-0.23865000	-0.40520500
C	0.61108500	1.41190200	-0.04168500
C	-0.70642800	1.11090600	-0.32423400
H	0.96949500	2.42497600	0.08083100
O	-1.70556300	-0.44112600	1.53434700
H	-2.11199100	-1.31489700	1.43833000
H	-2.09358400	-0.48492000	-0.81090500
O	-1.66185600	2.03399900	-0.48865100
H	-1.30622500	2.91938000	-0.35426600
O	2.77454600	0.60850000	0.35980600
H	3.25285500	-0.22940200	0.40816300
N	1.16986200	-0.94083300	-0.09592200

[HO-THP-OH]• (5-OH)

G = -549.606513 au

0 2	x	y	z
O	-1.75322900	-1.85944200	-0.53296600
H	-2.59062400	-1.40382000	-0.37374600
C	-0.75447900	-0.98087700	-0.38156200
C	1.50989500	-0.49116500	0.09371000
C	-1.00174800	0.40675800	-0.47151000
C	0.10884100	1.24909400	-0.28492700
O	-2.19903300	0.43468600	1.28852200
H	-1.50691000	0.22275100	1.93302800
H	-1.90237500	0.80081500	-0.91784000
O	-0.08471800	2.56375400	-0.41659700
H	0.75699800	3.00987400	-0.25505600
O	2.74464300	-0.91306200	0.38943800
H	3.30688900	-0.13566200	0.50203900
C	0.51556800	-1.44866600	-0.09651100
N	1.32597500	0.82721800	0.01702400

H	0.72443900	-2.50283300	0.01003400

[HO-THP-OH]$^{\bullet}$ (6-OH)

G = -549.593844 au

0 2	x	y	z
O	2.33898000	0.53122800	-0.77503800
H	2.90155900	-0.24458400	-0.65669400
C	1.12497700	0.22668700	-0.30768200
C	-1.19901000	0.86244400	-0.15266200
C	-1.50592200	-0.46925700	0.03533400
C	-0.44027600	-1.39299800	-0.03349300
H	-2.51967400	-0.80516300	0.19729100
O	1.36827900	0.84626400	1.51552800
H	0.61206900	0.49703800	2.00965800
O	-0.76018500	-2.68621600	0.10145900
H	0.05625700	-3.19987400	0.04212600
O	-2.20307600	1.76055100	-0.13223100
H	-1.85979100	2.65580400	-0.22177900
N	0.81968700	-1.08968700	-0.22824400
C	0.13440700	1.24641100	-0.36060900
H	0.43474300	2.27025000	-0.53599000

THB-O$^{\bullet}$ (1-OH, 3-OH, 5-OH)

G = -533.524951 au

0 2	x	y	z
H	2.36335400	-1.18180500	0.11556600
O	1.62959500	-1.81086800	-0.39991500
C	0.62113800	-0.98063400	-0.36532300
C	-0.69287400	-1.39972000	-0.16355700
C	0.93023700	0.42328300	-0.44351000
C	-1.66209300	-0.43600800	0.08161600
C	-0.08827600	1.36402900	-0.14347100
C	-1.37016600	0.94680500	0.12346500
O	2.74143600	-0.05753000	0.79011500
H	2.41107500	-0.06474500	1.69684000
H	-0.91680300	-2.45771300	-0.10833200
H	-2.17425900	1.64501700	0.32131600
H	1.79376000	0.75918100	-0.99812900
O	0.27621700	2.66085600	-0.20099900
H	-0.48281800	3.23138500	-0.03993000
O	-2.94182500	-0.75808500	0.33426900
H	-3.06549400	-1.71283800	0.28959500

[HO-THB-OH]$^{\bullet}$ (1-OH, 3-OH, 5-OH)

G = -533.518189 au

0 2	x	y	z
O	2.35516000	-0.64210400	-0.81980200
C	0.68265300	1.04634200	-0.37401900
H	3.00146300	0.01166700	-0.52765300
C	1.13979900	-0.30058400	-0.33208400
C	-0.66618900	1.31521300	-0.13868400
C	0.19771600	-1.35943900	-0.24183900
C	-1.57073600	0.28421400	0.04077100
C	-1.12151200	-1.05446800	-0.02860500
H	-2.62194200	0.47463100	0.22281700
O	1.80095800	0.05191200	1.49032900
H	1.04023300	0.31995200	2.02521600
H	0.55846000	-2.37888000	-0.29267800
H	1.38171700	1.85526400	-0.54141400
O	-2.08434900	-1.99701800	0.12609000
H	-1.69411600	-2.87625900	0.09120200
O	-1.02864400	2.62497800	-0.11275600
H	-1.98120500	2.70380600	-0.00161400

[HO-THB-OH]• (2-OH, 4-OH, 6-OH)

G = -533.529721 au

0 2	x	y	z
O	0.50438000	2.60680800	-0.36454300
C	-1.24612100	1.01798000	0.05374000
H	-0.21596100	3.22316300	-0.19739700
C	0.06609500	1.33348800	-0.25414100
C	-1.62111800	-0.32868600	0.09538500
C	1.02320600	0.32784700	-0.48385000
C	-0.70393500	-1.35412100	-0.12977100
C	0.61115900	-1.01547800	-0.41209900
H	-0.98715000	-2.39682100	-0.05504300
O	2.34399200	0.00289300	1.27619400
H	1.63056100	-0.05458400	1.92998800
H	1.98381400	0.59787900	-0.89836200
H	-1.99053000	1.78349000	0.23618400
O	1.50715000	-2.00821200	-0.58979300
H	2.36833300	-1.67367500	-0.30234900
O	-2.91621200	-0.58097300	0.38806500
H	-3.07926900	-1.52976500	0.39201400

Cartesian coordinates of equilibrium geometries (water)

THP-O⁻ (4-OH)

G = -473.429804 au

-1 1	x	y	z
C	-0.62229900	-1.13007700	-0.00000400
C	0.75165500	-1.20804400	0.00001500
C	1.52580600	0.00165500	0.00013200
C	0.74857800	1.20983200	0.00000400
C	-0.62495300	1.12891100	-0.00003500
N	-1.33987100	-0.00164700	0.00000100
H	1.24513600	-2.17154300	-0.00003600
H	1.24049400	2.17411100	-0.00004300
O	2.79180600	0.00332900	-0.00005600
O	-1.34429000	-2.28596600	-0.00001500
H	-2.27645600	-2.03605600	0.00000300
O	-1.35016600	2.28278200	-0.00000700
H	-2.28160400	2.03019000	0.00002300

THP-O$^{\bullet-}$ (3-OH, 5-OH)

G = -472.796213 au

-1 2	x	y	z
C	-0.28832700	1.39399100	-0.00011900
C	1.09123100	0.90315700	-0.00001400
C	1.41690100	-0.52022200	-0.00002200
C	0.28031800	-1.36142100	-0.00002300
C	-0.99323700	-0.78467000	-0.00000600
N	-1.31959100	0.50099500	-0.00002400
H	1.89184000	1.63314800	0.00005700
H	0.38233100	-2.43815100	0.00000000
O	2.61389300	-0.89642100	0.00004500
O	-0.48592500	2.62130900	0.00007000
O	-2.03273600	-1.64640300	0.00003100
H	-2.84020500	-1.11485100	0.00004100

THP-O^{2-} (3-OH, 5-OH)

G = -472.954209 au

-2 1	x	y	z
C	-0.22177600	1.39790800	0.00000100
C	1.10598700	0.89188500	-0.00002500
C	1.40232300	-0.49636800	-0.00015600
C	0.25736400	-1.36652700	-0.00002700
C	-0.99445200	-0.78686300	-0.00002000
N	-1.28130500	0.50853200	-0.00003200
H	1.92747000	1.60164500	0.00004500
H	0.37625800	-2.44291100	0.00004500
O	2.59864300	-0.96166700	0.00011000

O	-0.48267600	2.64864900	0.00004200
O	-2.08749800	-1.62601300	0.00003200
H	-2.85902100	-1.04642200	0.00002500

THP-O$^{\bullet}$ (4-OH)

G = -473.211214 au

0 2	x	y	z
C	-0.66878000	1.11217500	-0.00000400
C	0.75406400	1.18765100	0.00000200
C	1.55438900	0.00000300	-0.00001700
C	0.75406300	-1.18764700	0.00000200
C	-0.66877000	-1.11218400	-0.00000200
N	-1.39476700	0.00000000	-0.00000800
H	1.21522800	2.16724300	0.00001800
H	1.21526000	-2.16722300	0.00001800
O	2.80340300	-0.00000200	0.00000800
O	-1.30728100	2.25999000	0.00000400
H	-2.26380600	2.09529400	0.00000300
O	-1.30728500	-2.25999000	0.00000500
H	-2.26381000	-2.09528800	0.00000300

[HO-THB-OH]$^{\bullet-}$ (2-C)

G = -549.182448 au

-1 2	x	y	z
C	-0.55673700	1.28542700	-0.00015700
C	-1.61539100	0.34388200	0.00018400
C	-1.32481000	-1.03544600	-0.00022800
C	0.09083200	-1.34834400	-0.00089700
C	1.15471400	-0.29428100	-0.00007800
N	0.72352000	1.06989500	-0.00054100
H	-2.63675100	0.69996500	0.00059700
H	0.41385500	-2.38293100	-0.00038400
O	-2.18349500	-1.96463700	-0.00013700
O	-0.95123200	2.58628100	-0.00006800
H	-0.14812900	3.12336200	0.00003000
O	1.95245200	-0.51778600	-1.15487400
H	2.33620800	0.33754600	-1.38383800
O	1.95025900	-0.51783800	1.15622800
H	2.33466900	0.33719300	1.38525200

[HO-THB-OH]$^{\bullet-}$ (3-C, 5-C)

G = -549.182978 au

-1 2	x	y	z
C	-1.53721000	-0.21958800	0.07084600

C	-0.75270900	-1.36871600	-0.05382600
C	0.63480900	-1.26532800	-0.27090900
C	1.24588900	0.12474400	-0.14683400
C	0.21087800	1.18126500	-0.24082500
N	-1.08748900	1.02933000	-0.04623100
H	-1.22280900	-2.34427200	-0.03228800
O	1.41255700	-2.23204400	-0.49945300
O	-2.87795100	-0.36815300	0.29425600
H	-3.26152000	0.51673300	0.31341200
O	0.69082600	2.44080800	-0.45779700
H	-0.06537500	3.04060400	-0.47739900
H	2.01519100	0.26299900	-0.91322500
O	2.01565500	0.23038800	1.09586600
H	1.40829800	0.03637100	1.81942800

[HO-THB-OH]$^{\bullet-}$ (4-C)

G = -549.150439 au

-1 2	x	y	z
C	-0.87509800	1.14589900	-0.00445700
C	0.46421200	1.26408500	-0.01055900
C	1.34621800	0.03953200	-0.08724100
C	0.52243800	-1.19822300	0.10595200
C	-0.87469100	-1.14115900	0.04796900
N	-1.58147900	-0.03799100	-0.00161800
H	0.93312300	2.24076500	-0.03719100
O	1.93532600	-0.15940300	-1.27758200
O	-1.67723500	2.25505100	-0.01274400
H	-2.58928500	1.94378500	0.01667700
O	-1.54971300	-2.32054600	0.07255300
H	-2.49303100	-2.11362500	0.08435300
H	1.03610200	-2.15035100	0.13449200
O	2.38344300	0.14039100	0.93471800
H	1.95040900	0.36061300	1.76744600

THB-OH (1-OH, 3-OH, 5-OH)

G = -457.806941 au

0 1	x	y	z
C	-1.17803600	-0.76057000	0.00001600
C	-1.23122300	0.62796600	0.00000500
C	-0.06964300	1.40026300	0.00000700
C	1.15955800	0.75231200	0.00000200
C	1.24704000	-0.63957700	-0.00001000
C	0.07123000	-1.38015600	0.00000300
H	-2.07864200	-1.36271900	0.00003700

H	-0.14115300	2.48117800	-0.00001900
H	2.21824600	-1.11917800	-0.00005700
O	2.33480600	1.43627000	0.00000200
H	2.16779200	2.38474500	0.00002700
O	-2.41147600	1.30333000	-0.00001400
H	-3.14851400	0.68357500	0.00000200
O	0.07739000	-2.73979700	-0.00001200
H	0.98295000	-3.06744800	0.00006300

THB-O$^{\bullet}$ (1-OH, 3-OH, 5-OH)

G = -457.169513 au

0 2	x	y	z
C	1.30967300	0.62601900	0.00000500
C	1.15633800	-0.73301100	0.00000500
C	-0.13339500	-1.31605800	0.00000300
C	-1.28387200	-0.50600500	-0.00000300
C	-1.16907200	0.86210900	-0.00000600
C	0.13969700	1.48173000	0.00000400
H	2.28646200	1.09531800	0.00000900
H	-0.21508900	-2.39760100	0.00000000
H	-2.04628100	1.49660700	-0.00001900
O	-2.52491000	-1.04914100	-0.00000500
H	-2.47804100	-2.01126300	0.00000500
O	2.18322900	-1.61445000	0.00000600
H	3.02734600	-1.14903300	0.00001700
O	0.25535400	2.72325000	-0.00000900

[HO-THP-OH]$^{\bullet}$ (1-OH, 3-OH, 5-OH)

G = -533.557405 au

0 2	x	y	z
C	-0.43895400	-1.25177400	-0.01620700
C	-1.26419900	0.00265300	-0.01412400
C	-0.44894000	1.26004700	-0.02487100
C	0.90368700	1.23017500	-0.01007200
C	1.62526400	0.00226900	-0.00454000
C	0.92067200	-1.22317300	-0.01140600
H	-0.97304700	-2.19574000	-0.01367500
H	-1.00496700	2.19091600	-0.03178100
H	2.70822800	0.02897400	0.00449400
O	1.68982600	2.34305300	0.00155500
H	1.14119400	3.13515300	-0.01239000
O	-2.11837200	0.05580400	-1.13569400
H	-2.69591100	-0.71741300	-1.10480300
O	1.59463600	-2.40921700	-0.00949000

H	2.54507500	-2.25639700	-0.00114900
O	-2.15104000	-0.00977900	1.11092900
H	-1.62614500	-0.14557000	1.90821700

[HO-THP-OH]• (2-OH, 4-OH, 6-OH)

G = -533.560359 au

0 2	x	y	z
C	-1.01987800	1.20009600	-0.00619500
C	0.33383000	1.24975900	-0.15539300
C	1.19819100	0.03567400	-0.26049900
C	0.38923400	-1.22017400	-0.18343800
C	-0.96544300	-1.25077100	-0.03513100
C	-1.68578400	-0.04293000	0.04941200
H	-1.60380400	2.11220300	0.05438700
H	1.76470300	0.06540600	-1.20342600
H	-1.47402900	-2.20665900	0.02773500
O	1.09490200	-2.37742300	-0.28417700
H	2.02178600	-2.19057000	-0.08625300
O	1.05030100	2.39796700	-0.26625700
H	0.46835000	3.16332200	-0.19658600
O	-3.03619000	-0.00980300	0.21228600
H	-3.39189500	-0.90467500	0.23015300
O	2.25061900	0.00211200	0.72826200
H	1.83692700	0.02821800	1.60053500

THB-O⁻ (1-OH)

G = -457.343138 au

-1 1	x	y	z
C	1.31986600	0.51428800	0.00000800
C	1.05148700	-0.84340700	0.00000700
C	-0.25155100	-1.34828300	0.00000800
C	-1.29275700	-0.41764500	-0.00000200
C	-1.06814800	0.94887100	-0.00000400
C	0.25979900	1.48104900	0.00001400
H	2.34647700	0.86966400	0.00000600
H	-0.43137300	-2.41645800	0.00000200
H	-1.91260100	1.62897500	-0.00002300
O	-2.60162000	-0.83552700	-0.00001000
H	-2.62995400	-1.79744600	0.00000100
O	2.06108100	-1.77451000	0.00000400
H	2.90949200	-1.32002000	0.00000900
O	0.49126200	2.73829300	-0.00001700

THB-O•• (3-OH, 5-OH)

G = -456.534873 au

0 3	x	y	z
C	0.54358200	-1.27730900	0.00001100
C	-0.90033300	-1.18424500	0.00001800
C	-1.48483700	0.14185300	-0.00000200
C	-0.65186500	1.32870400	-0.00000800
C	0.78553500	1.13767500	-0.00000400
C	1.34378800	-0.13265200	0.00000200
H	0.99831100	-2.25995700	0.00001300
H	-2.56207500	0.24912400	-0.00000900
H	1.40776600	2.02570900	-0.00001000
O	-1.15862900	2.45992200	-0.00002300
O	-1.61819600	-2.19676000	0.00000500
O	2.67514000	-0.33790200	0.00000500
H	3.15426100	0.49888700	-0.00000100

THB-O$^{\cdot -}$ (3-OH, 5-OH)

G = -456.717656 au

-1 2	x	y	z
C	0.48667900	-1.27907600	-0.00000600
C	-0.97803800	-1.11996800	-0.00008200
C	-1.48478900	0.19604200	-0.00001800
C	-0.63825800	1.32792400	0.00000400
C	0.81932400	1.09461700	-0.00001700
C	1.34131600	-0.18157000	-0.00000200
H	0.88914200	-2.28546700	0.00002900
H	-2.55805800	0.34763600	0.00000900
H	1.46254700	1.96886700	-0.00001100
O	-1.03577800	2.52343400	0.00000400
O	-1.67970700	-2.16886900	0.00005800
O	2.68486400	-0.43610900	0.00002200
H	3.17392900	0.39350800	0.00002800

HO-THBO (2-C)

G = -533.003737 au

0 1	x	y	z
C	-1.09686200	1.24653300	-0.05882800
C	0.32872600	1.33753500	-0.02648800
C	1.08967800	0.06759300	-0.40388200
C	0.38940900	-1.20390500	-0.02636700
C	-0.95200200	-1.23891300	0.05718600
C	-1.68281500	0.00957000	-0.01652900
H	-1.70052100	2.14044100	0.02172300
H	1.08061400	0.07814800	-1.50938400
H	-1.47938300	-2.16642600	0.24202000

O	1.15292400	-2.28420200	0.12895300
H	2.08087500	-2.00654900	0.17460700
O	0.99554800	2.33702900	0.21816500
O	-3.01988600	-0.04032300	0.02590300
H	-3.33543600	-0.95030700	0.07516100
O	2.40747600	0.06475000	0.06654300
H	2.60855900	0.97618800	0.32879900

HO-THBO (4-C)

G = -533.001477 au

0 1	x	y	z
C	-0.98679300	-1.32020900	0.00246600
C	-1.79980300	-0.10221200	0.04718000
C	-1.10778900	1.19438800	0.02003400
C	0.22431700	1.26881400	-0.10442100
C	1.08650400	0.05407500	-0.32133700
C	0.34506200	-1.24498200	-0.11227200
H	-1.48917200	-2.27388200	0.10458900
H	-1.71972600	2.08464800	0.11230800
H	1.39946700	0.07393800	-1.37353300
O	0.95133100	2.39513500	-0.11763900
H	0.38349100	3.16740100	-0.00345300
O	-3.02192000	-0.15688100	0.13339600
O	1.12091300	-2.33786100	-0.10488300
H	2.04804900	-2.06870800	-0.02905600
O	2.28964800	0.06499800	0.41965700
H	2.08912600	0.19424100	1.35499700

HO-THBO (6-C)

G = -533.002103 au

0 1	x	y	z
C	-1.13210400	0.07134700	-0.36411600
C	-0.32394500	1.33802600	-0.05927800
C	1.10219200	1.23080000	-0.07353700
C	1.66815800	-0.01124700	0.00160700
C	0.91757200	-1.24671600	0.05980500
C	-0.42286800	-1.20918400	-0.03797600
H	1.46450400	-2.17108500	0.19696000
O	2.98998400	-0.20265100	0.07047500
H	3.46006400	0.63999000	0.03906900
O	-0.96279000	2.36670800	0.13301200
O	-1.24049300	-2.26254900	0.03097800
H	-0.74076200	-3.07569300	0.17866500
O	-2.40515000	0.13437100	0.21252900

H	-2.58610300	1.07915500	0.32975700
H	1.70237800	2.13058400	-0.01095400
H	-1.20652300	0.07185700	-1.46848300

THB=O

G = -456.500638 au

0 1	x	y	z
C	1.52107700	0.25927500	-0.00001300
C	0.59861900	1.34152400	0.00000100
C	-0.89296200	1.06919100	0.00000200
C	-1.36993700	-0.20688100	0.00001200
C	-0.40727400	-1.23255200	0.00000600
C	1.07105400	-1.06173700	-0.00002100
H	2.57883300	0.48528500	-0.00001700
H	-1.53824100	1.94179600	0.00000500
H	-0.75944000	-2.26252600	0.00001000
O	-2.65882100	-0.58671100	0.00002400
H	-3.25032000	0.17599100	0.00002700
O	0.89904400	2.53277400	-0.00001400
O	1.74049000	-2.11524600	-0.00000300

Cartesian coordinates of transition states (water)

THP-O⁻ (2-OH, 6-OH)

G = -549.167105 au

-1 2	x	y	z
H	-2.24346800	-1.37140000	-0.00812100
O	-1.47728400	-1.86268900	-0.45489700
C	-0.52307700	-0.93480100	-0.36522600
C	-0.93782100	0.42909500	-0.47431100
C	1.60729300	-0.32416600	0.10083200
C	0.01921600	1.48207400	-0.16641100
O	-2.67345500	-0.07686800	0.85732600
H	-2.24849700	-0.09551400	1.72284400
O	-0.30218000	2.68870300	-0.21649300
O	2.86820400	-0.75242900	0.32393200
H	2.85716100	-1.71753800	0.28766600
H	-1.80848900	0.68182700	-1.05999400
C	1.34330700	1.01884500	0.14434700
N	0.70500000	-1.31781600	-0.11636500
H	2.13249300	1.72731700	0.35783800

[HO-THB-OH]•⁻ (2-C, 4-C)

G = -549.157385 au

-1 2	x	y	z
O	-2.38868900	-0.29510700	-0.73087200
H	-2.61119000	-1.23112400	-0.64857400
C	-1.11873100	-0.15064600	-0.30152900
C	0.92134000	-1.09110000	-0.03795300
C	-0.55277100	1.15504300	-0.40013600
C	1.58762500	0.12130200	0.05879600
C	0.86789700	1.33320500	-0.16242000
H	2.65443800	0.14079000	0.23577100
O	-1.50568500	0.34558900	1.54321700
H	-0.63922000	0.30722900	1.96901300
H	-1.19102400	2.00666900	-0.59143400
O	1.38313700	2.47864700	-0.16962100
O	1.65711600	-2.22041100	0.08912100
H	1.04865400	-2.96720100	0.01947000
N	-0.38012100	-1.27613300	-0.25422200

THB-O$^{\bullet}$ (1-OH, 3-OH, 5-OH)

G = -533.533694 au

0 2	x	y	z
H	2.35373000	-1.19965400	0.10541200
O	1.63321000	-1.81612500	-0.40999400
C	0.61513500	-0.98434900	-0.36816200
C	-0.69125900	-1.40182200	-0.15934100
C	0.92879400	0.41727800	-0.45141800
C	-1.66218100	-0.43097300	0.08256100
C	-0.08491100	1.36444400	-0.14804100
C	-1.36730100	0.95119100	0.12037400
O	2.72589700	-0.05081500	0.81252400
H	2.36121100	-0.07685600	1.70593800
H	-0.92289000	-2.45751600	-0.09666400
H	-2.16774500	1.65350400	0.31797200
H	1.78809000	0.74556300	-1.01699800
O	0.28893000	2.65672800	-0.20471500
H	-0.46426600	3.23707700	-0.04315300
O	-2.93673600	-0.75106300	0.33645200
H	-3.06818300	-1.70652300	0.29752100

[HO-THB-OH]$^{\bullet}$ (1-C, 3-C, 5-C)

G = -533.526487 au

0 2	x	y	z
O	2.36011400	-0.65222500	-0.80745300
C	0.69560700	1.04397600	-0.37041100

H	2.98059100	0.07389700	-0.67118300
C	1.14500900	-0.30590300	-0.33023300
C	-0.65386100	1.31950300	-0.14176600
C	0.19870500	-1.36256700	-0.24450800
C	-1.56309800	0.29255600	0.03278700
C	-1.11955400	-1.04993700	-0.03585000
H	-2.61318900	0.49065800	0.21227300
O	1.75879700	0.04211900	1.52561600
H	0.95380400	0.22099300	2.03239700
H	0.55015100	-2.38510800	-0.29861500
H	1.40021800	1.84945300	-0.53315900
O	-2.08819300	-1.98560300	0.11216400
H	-1.70847500	-2.87048500	0.07278500
O	-1.00816800	2.63031300	-0.11503200
H	-1.96034600	2.71799400	0.00303100

[HO-THB-OH]$^{\bullet}$ (2-C, 4-C, 6-C)

G = -533.628309 au

0 2	x	y	z
O	0.90011067	2.46267377	-0.49538395
C	-1.05584093	1.14698497	-0.03583966
H	0.27731589	3.18183696	-0.34053248
C	0.28822813	1.26609835	-0.34760308
C	-1.60536830	-0.13381663	0.07889559
C	1.10108964	0.13218044	-0.51286564
C	-0.83613051	-1.28116410	-0.09971205
C	0.51111349	-1.13488356	-0.40716760
H	-1.26401905	-2.27111045	0.00113445
O	2.03830528	0.00064248	1.59746386
H	1.15731352	-0.02723596	2.00376193
H	2.12350600	0.25051511	-0.84624118
H	-1.68590139	2.01592485	0.10831739
O	1.23582271	-2.26258204	-0.57445184
H	2.16822171	-2.04333797	-0.68182887
O	-2.92211537	-0.19641233	0.38002747
H	-3.21197208	-1.11356432	0.43989905
